# Supplementary material for: Hierarchical communication of chirality for aromatic oligoamide sequences
Source: Nat Commun. 2021 May 11;12:2659. doi: 10.1038/s41467-021-22984-6 (PMC8113567; doi:10.1038/s41467-021-22984-6)
Supplement: Supplementary file 1 — Supplementary Information [file 41467_2021_22984_MOESM1_ESM.pdf]

Supplementary Information for

Hierarchical Communication of Chirality for Aromatic Oligoamide Sequences

Jiajia Zhang, Dan Luo, Chunmiao Ma, Lu Huang and Quan Gan*

Hubei Key Laboratory of Bioinorganic Chemistry & Materia Medica, Hubei Engineering Research Center for Biomaterials and Medical Protective Materials, School of Chemistry and Chemical Engineering, Huazhong University of Science and Technology, Wuhan, 430074, P. R. China

Correspondence and requests for materials should be addressed to Quan Gan (ganquan@hust.edu.cn).

Table of contents

1 Experimental procedures 3

1.1 General methods 3

1.2 Synthesis of Ligand **L** 4

1.3 Synthesis of Ligand $\mathbf{L}^{R}$ and $\mathbf{L}^{S}$ 4

2 Solution spectroscopic analysis of the helicates 1 and 2 8

2.1 Self-assembly of the monomeric helicate **1** and dimeric helicate **2** 8

2.2 Dynamic exchange between the monomeric helicate **1** and dimeric helicate **2** 11

2.3 Full characterization of the dimeric helicate **2** 14

3 Method for definition of P/M helicity 16

4 Solution spectroscopic analysis of the helicate 1-Pt 18

4.1 Self-assembly of the monomeric helicate **1-Pt** 18

4.2 The *P*/*M* helical interconversion of helicate **1-Pt** induced by anion guests 19

5 Molecular Modelling of 2 21

6 Solution spectroscopic analysis of the chloride complex [2Cl⊂2] 24

6.1 Self-assembly of the complex [2Cl⊂**2**] 24

6.2 Capturing and releasing chloride anions within the dimeric helicate 27

7 X-Ray Crystallography 28

8 Solution spectroscopic analysis of the helicates 3R **and 4**R 31

8.1 Self-assembly of the monomeric helicate **3**R and dimeric helicate **4**R (or **3**S/**4**S) 31

8.2 Self-assembly of the complexes [2Cl⊂**4**R] and [2Cl⊂**4**S] 36

8.3 Amplification of chirality of the dimeric helicates 38

9 Supplementary NMR spectra 39

10 Supplementary References 51

1 Experimental procedures

1.1 General methods

All chemicals and solvents were purchased from commercial suppliers and were used without further purification unless otherwise specified. Dichloromethane (DCM) and *N*,*N*-diisopropylethylamine (DIEA) was distilled over CaH_2 prior to use. Column chromatography was carried out on Merck GEDURAN Si60 (40-63 μm).

NMR spectra were recorded on Bruker AVANCE 600 (600 MHz), and Bruker AVANCE 400 (400 MHz) spectrometers. The solvent signals were assigned by Fulmer et al.[1] All chemical shifts (δ) are quoted in ppm and coupling constants (J) are expressed in Hertz (Hz). The following abbreviations are used for convenience in reporting the multiplicity for NMR resonances: s = singlet, d = doublet, t = triplet, and m = multiplet. Data processing was performed with Topspin 2.0 software. Assignment of all ^{1}H and ^{13}C resonances was achieved using standard 2D NMR techniques: ^{1}H-^{1}H COSY, and ^{1}H-^{1}H NOESY.

High-resolution electrospray ionization mass spectrometry (ESI-MS) was performed on a micro TOF II instrument featuring a Z spray source with electrospray ionization and modular LockSpray interface.

CD studies were recorded using a 2 mm pathlength cell on a Jasco J-810 spectropolarimeter.

1.2 Synthesis of Ligand **L**

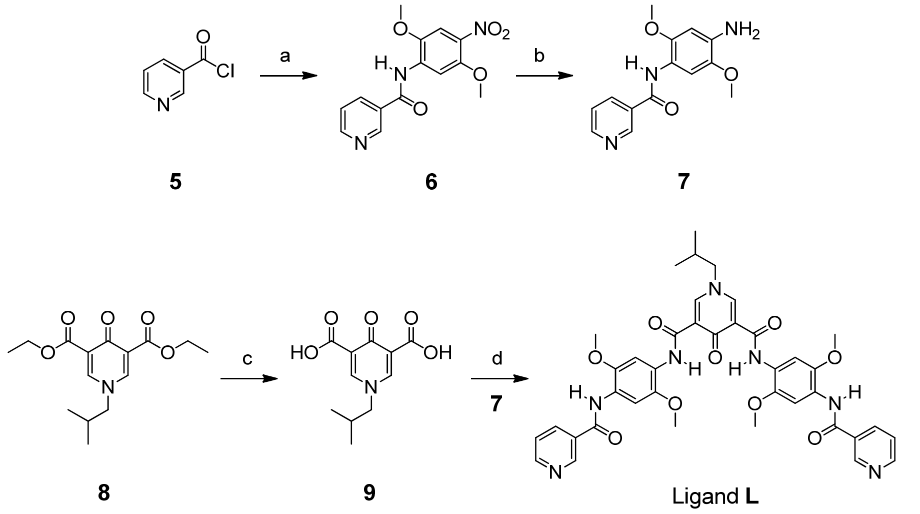

Supplementary Scheme 1. Synthesis of Ligand **L**. a) 2,5-dimethoxy-4-nitrobenzenamine, dry DIEA, dry DCM, r.t., 2 days, 79%; b) Pd/C, DCM, r.t., 20 hours, 59%; c) NaOH, ethanol, H_2O, r.t., 5 hours, 90%; d) (benzotriazol-1-yloxy)tripyrrolidinophosphonium hexafluorophosphate, dry DIEA, dry DMF, r.t., 2 days, 69%.

1.3 Synthesis of Ligand $\mathbf{L}^R$ and $\mathbf{L}^S$

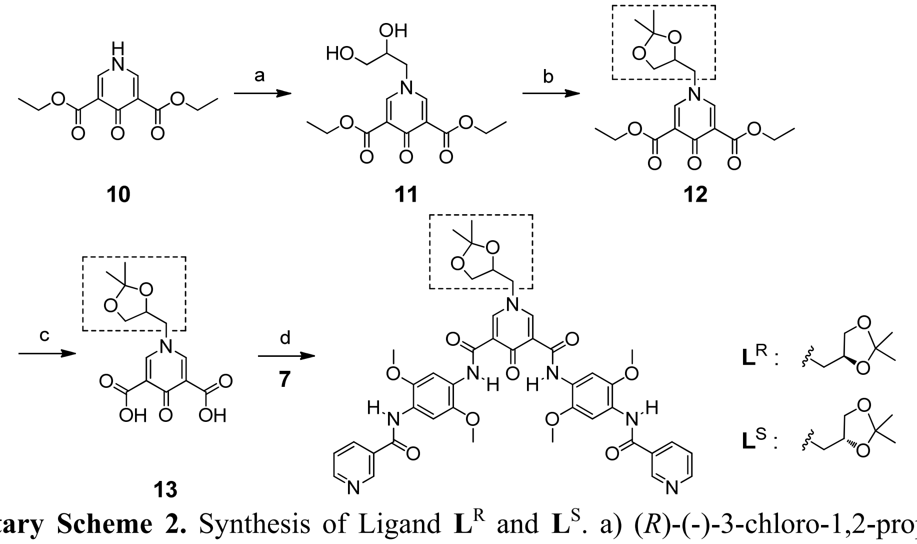

Supplementary Scheme 2. Synthesis of Ligand $\mathbf{L}^R$ and $\mathbf{L}^S$. a) (*R*)-(-)-3-chloro-1,2-propanediol (or (*S*)-(+)-3-chloro-1,2-propanediol), K_2CO_3, dry DMF, 70 °C, 18 hours, 56%; b) *p*-toluenesulfonic acid, acetone, ethanol, 65 °C, 2 days, 97%; c) NaOH, ethanol, H_2O, r.t., overnight, 89%; d) (benzotriazol-1-yloxy)tripyrroli-dinophosphonium hexafluorophosphate, dry DIEA, dry DMF, r.t., 2 days, 75%.

Compound **5** was prepared accordingly to Supplementary Reference 2. Compound **8** and compound **10** were prepared accordingly to Supplementary Reference 3.

Compound **6**. To a suspension of 2,5-dimethoxy-4-nitrobenzenamine (3.96 g, 20 mmol) and compound **5** (2.83 g, 20 mmol) in dry DCM (60 mL) under nitrogen, dry DIEA (17.47 ml, 100 mmol) were added slowly. The reaction mixture was stirred at room temperature for 2 days. After that time, the reaction mixture was poured into 300ml petroleum ether to effect precipitation, the precipitate was obtained by filtration. And it was then washed with water and ice-cold MeOH, dried and desiccated to yield compound **6** as a pale yellow powder (4.79 g, 79%). ^{1}H NMR (DMSO-d_6, 400 MHz): δ 10.05 (s, 1 H), 9.10 (s, 1 H), 8.79 (d, J = 3.88 Hz, 1 H), 8.29 (d, J = 7.80 Hz, 1 H), 8.09 (s, 1 H), 7.68 (s, 1 H), 7.57 (q, J = 4.92 Hz, 1 H), 3.90 (s, 6 H). ^{13}C NMR (DMSO-d_6, 100 MHz): δ 164.5, 152.6, 148.8, 147.3, 143.6, 135.6, 134.0, 133.1, 129.7, 123.6, 108.2, 56.9, 56.7. ESI-HRMS: m/z calcd for $C_{14}H_{13}N_3O_5$ $[M+H]^+$ 304.0928, found 304.0980.

Compound **7**. Compound **6** (2.00 g, 6.60 mmol) was dissolved in DCM (60 mL) and 10% Pd/C (10% by mass) was added. The reaction mixture was stirred under H_2 for 20 hours (TLC, 90 : 10 DCM : MeOH), then was filtered through a pad of celite and washed with methanol. The solvent was evaporated and the crude product was purified by column chromatography (from DCM : MeOH 98 : 2 to DCM : MeOH 90 : 10) to afford compound **7** as a light yellow green oil (1.06 g, 59%). ^{1}H NMR ($CDCl_3$, 400 MHz): δ 9.10 (s, 1 H) ,8.76 (d, J = 4.48 Hz, 1 H), 8.36 (s, 1 H), 8.20 (d, J = 8.08 Hz, 1 H), 8.14 (s, 1 H), 7.44 (q, J = 4.56 Hz, 2 H), 6.40 (s, 1 H), 3.89 (s, 3 H), 3.85 (s, 3 H), 3.79 (s, 2 H). ^{13}C NMR ($CDCl_3$, 100 MHz): δ 162.6, 152.1, 147.9, 143.1, 140.6, 134.9, 132.9, 131.1, 123.6, 118.0, 104.9, 99.0, 56.3, 56.2. ESI-HRMS: m/z calcd for $C_{14}H_{15}N_3O_3$ $[M+H]^+$ 274.1186, found 274.1178.

Compound **9**. Compound **8** (0.63 g, 2.14 mmol) was dissolved in ethanol/H_2O (10 mL/2 mL), to which KOH (1.20 g, 21.40 mmol) was added in the surrounding atmosphere. The mixture was allowed to stir at room temperature for overnight. Then ethanol was removed in vacuo and the aqueous layer was brought to pH 3 ~ 4 by slow addition of 1 M HCl aqueous solution. The resulting mixture was extracted three times with EtOAc, and the combined organic phases, which washed by saturated NaCl aqueous solution and dried over anhydrous $MgSO_4$ and evaporated to yield compound **9** as a white solid (0.46 g, 90%), which was directly used in the next step without further purification. ^{1}H NMR ($CDCl_3$, 400 MHz): δ 13.50 (s, 2 H), 8.58 (s, 2 H), 3.92 (d, J = 7.52 Hz, 2 H), 2.24 - 2.15 (m, 1 H), 1.03 (d, J = 6.60 Hz, 6 H). ^{13}C NMR ($CDCl_3$, 100 MHz): δ 178.0, 163.9, 147.7, 119.0, 66.8, 30.3, 19.4. ESI-HRMS: m/z calcd for $C_{11}H_{13}NO_5$ $[M+Na]^+$ 262.0686, found 262.0667.

Ligand **L**. Compound **9** (0.15 g, 0.64 mmol), compound **7** (0.37 g, 1.34 mmol) and (benzotriazol-1-yloxy)tripyrrolidinophosphonium hexafluorophosphate (0.83 g, 1.60 mmol) was dissolved in dry DMF (10 mL) under nitrogen, dry DIEA (0.55 ml, 3.2 mmol) was added. The reaction mixture was stirred at room temperature for 2 days. Then the reaction mixture was poured into 150ml water to effect precipitation. The precipitate was collected by filtration, which was purified by washing with water,

ice-cold MeOH and CH_3CN, dried and desiccated to yield compound **L**[1] as a yellow powder (0.33 g, 69%). ^{1}H NMR (DMSO-d$_6$, 400 MHz): δ 12.54 (s, 2 H) ,9.82 (s, 2 H), 9.13 (s, 2 H), 8.87 (s, 2 H), 8.76 (d, J = 3.80 Hz, 2 H), 8.47 (s, 2 H), 8.30 (d, J = 7.96 Hz, 2 H), 7.59 (s, 2 H), 7.55 (q, J = 5.08 Hz, 2 H), 4.16 (d, J = 6.80 Hz, 2 H), 3.96 (s, 6 H), 3.81 (s, 6 H), 2.16 - 2.09 (m, 1 H), 0.91 (d, J = 6.52 Hz, 6 H). ^{13}C NMR (DMSO-d$_6$, 100 MHz): δ 175.3, 163.8, 161.1, 152.1, 148.7, 146.4, 145.6, 142.0, 135.3, 130.1, 125.7, 123.5, 121.7, 120.8, 108.9, 104.5, 63.9, 56.8, 56.1, 29.5, 18.9. ESI-HRMS: m/z calcd for $C_{39}H_{39}N_7O_9$ $[M+H]^+$ 750.2882, found 750.2873.

Compound **11**. Compound **10** (2.39 g, 10.00 mmol) was dissolved in dry DMF (10 mL), to which anhydrous K_2CO_3 (6.90 g, 50.00 mmol) and (*R*)-(-)-3-chloro-1,2-propanediol (or (*S*)-(+)-3-chloro-1,2-propanediol) (8.40 mL, 100.00 mmol) were added. The mixture was heated at 70 °C for 18 hours. The reaction mixture was then filtered and the solvent was removed in vacuo. The residue was purified by column chromatography (from DCM : MeOH 98 : 2 to DCM : MeOH 90 : 10) to afford compound **11** as a white powder (1.75 g, 56%). ^{1}H NMR (DMSO-d$_6$, 400 MHz): δ 8.20 (s, 2 H), 5.24 (d, J = 5.40 Hz, 1 H), 4.84 (t, J = 5.36 Hz, 1 H), 4.18 (q, J = 7.08 Hz, 4 H), 4.15 - 4.11 (m, 1 H), 3.91 - 3.83 (m, 1 H), 3.75 - 3.67 (m, 1 H), 3.44 - 3.37 (m, 1 H), 3.29 - 3.22 (m, 1 H), 1.25 (d, J = 7.04 Hz, 6 H). ^{13}C NMR (DMSO-d$_6$, 100 MHz): δ 170.2, 164.2, 145.9, 121.3, 70.3, 62.8, 60.3, 59.2, 14.2. ESI-HRMS: m/z calcd for $C_{14}H_{19}NO_7$ $[M+H]^+$ 314.1234, found 314.1207.

Compound **12**. Compound **11** (1.00 g, 3.20 mmol) was dissolved in acetone/ethanol (50 mL/5 mL), to which *p*-toluenesulfonic acid (152 mg, 0.08 mmol) was added in the surrounding atmosphere. The mixture was heated at 65 °C for 2 days. Removal of the solvent in vacuo gave the crude product, which was purified by column chromatography (DCM : MeOH 90 : 10 as a eluent) to afford compound **12** as a white powder (1.09 g, 97%). ^{1}H NMR ($CDCl_3$, 400 MHz): δ 8.09 (s, 2 H), 4.44 - 4.31 (m, 5 H), 4.19 - 4.13 (m, 1 H), 4.04 - 3.97 (m, 1 H), 3.90 - 3.80 (m, 1 H), 3.73 - 3.67 (m, 1 H), 1.44 (s, 3 H), 1.35 (m + s, 9 H). ^{13}C NMR ($CDCl_3$, 100 MHz): δ 171.3, 164.8, 145.7, 122.9, 110.9, 74.2, 66.0, 61.4, 59.6, 26.7, 25.1, 14.4. ESI-HRMS: m/z calcd for $C_{17}H_{23}NO_7$ $[M+H]^+$ 353.1475, found 353.2644.

Compound **13**. Compound **12** (0.47 g, 1.33 mmol) was dissolved in ethanol/H_2O (10 mL/2 mL), to which NaOH (0.53 g, 13.30 mmol) was added in the surrounding atmosphere. The mixture was allowed to stir at room temperature for overnight. Then ethanol was removed in vacuo and the aqueous layer was brought to pH 5 ~ 6 by slow addition of 1 M HCl aqueous solution. The resulting mixture was extracted three times with DCM, and the combined organic phases, which washed by saturated NaCl aqueous solution and dried over anhydrous $MgSO_4$ and evaporated to yield compound **13** as a white solid (0.35 g, 89%), which was directly used in the next step without further purification. ^{1}H NMR ($CDCl_3$, 400 MHz): δ 13.47 (s, 2 H), 8.69 (s, 2 H), 4.51 - 4.43 (m, 1 H), 4.34 - 4.28 (m, 1 H),

4.27 - 4.21 (m, 1 H), 4.12 - 4.04 (m, 1 H), 3.80 - 3.74 (m, 1 H), 1.47 (s, 3 H), 1.34 (s, 3 H). ^{13}C NMR (DMSO-d_6, 100 MHz): δ 176.5, 164.2, 148.6, 118.2, 109.4, 73.8, 65.3, 59.1, 26.2, 24.9. ESI-HRMS: m/z calcd for $C_{13}H_{15}NO_7$ $[M+Na]^+$ 320.0741, found 320.0801.

Ligand $\mathbf{L}^R$ (or $\mathbf{L}^S$). Compound **13** (0.17 g, 0.58 mmol), compound **7** (0.35 g, 1.28 mmol) and (benzotriazol-1-yloxy)tripyrrolidinophosphonium hexafluorophosphate (0.76 g, 1.46 mmol) was dissolved in dry DMF (10 mL) under nitrogen, dry DIEA (0.51 ml, 2.90 mmol) was added. The reaction mixture was stirred at room temperature for 2 days. Then the reaction mixture was poured into 150ml water to effect precipitation. The precipitate was collected by filtration, which was purified by washing with water, ice-cold MeOH and CH_3CN, dried and desiccated to yield compound $\mathbf{L}^R$ (or $\mathbf{L}^S$) as a yellow powder (0.35 g, 75%). ^1H NMR (DMSO-d_6, 400 MHz): δ 12.52 (s, 2 H) ,9.82 (s, 1 H), 9.13 (s, 2 H), 8.89 (s, 2 H), 8.76 (d, J = 4.28 Hz, 2 H), 8.46 (s, 2 H), 8.31 (d, J = 7.36 Hz, 2 H), 7.59 (s, 2 H), 7.55 (q, J = 5.24 Hz, 2 H), 4.61 - 4.46 (m, 2 H), 4.44 - 4.35 (m, 1 H), 4.16 - 4.09 (m, 1 H), 3.96 (s, 6 H), 3.82 (s, 6 H), 3.77 - 3.71 (m, 1 H), 1.34 (s, 3 H), 1.28 (s, 3 H). ^{13}C NMR (DMSO-d_6, 100 MHz): δ 175.4, 163.8, 161.0, 152.1, 148.7, 147.1, 145.5, 142.0, 135.3, 130.1, 125.5, 123.5, 121.8, 120.6, 109.4, 108.9, 104.5, 74.1, 65.4, 59.3, 56.8, 56.1, 26.4, 25.0. ESI-HRMS: m/z calcd for $C_{41}H_{41}N_7O_{11}$ $[M+H]^+$ 808.2937, found 808.2870.

2 Solution spectroscopic analysis of the helicates 1 and 2

2.1 Self-assembly of the monomeric helicate **1** and dimeric helicate **2**

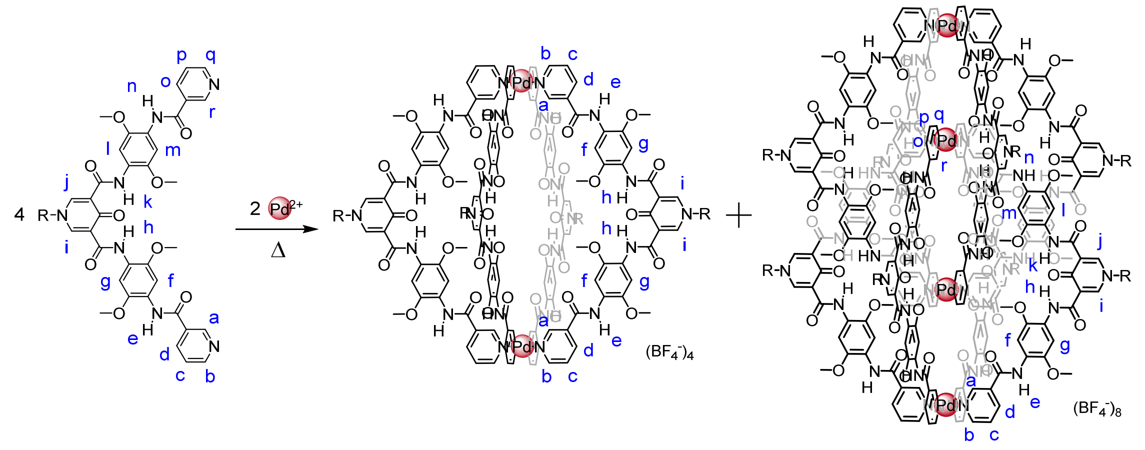

Ligand L: R = *iso*-butyl **1**: Pd_2L_4 **2**: Pd_4L_8

To a NMR tube were added ligand **L** (1.05 mg, 1.40 μmol), $Pd(CH_3CN)_4(BF_4)_2$ (0.31 mg, 0.70 μmol) and CD_3CN (500 μL). The reaction mixture was placed at 56 °C for 12 h to give a mixture solution of monomeric helicate **1** and dimeric helicate **2**. ^1H NMR (CD_3CN, 400 MHz): δ (for monomeric helicate **1**) 12.62 (s, 8 H), 9.93 (s, 8 H), 9.22 (d, *J* = 5.24 Hz, 8 H), 8.78 (s, 8 H), 8.63 (s, 8 H), 8.52 (s, 8 H), 8.42 (s, 8 H), 8.36 (d, *J* = 7.76 Hz, 8 H), 7.71 (t, *J* = 7.04 Hz, 8 H), 4.13 (s, 24 H), 3.96 (d, *J* = 7.32 Hz, 8 H), 3.87 (s, 24 H), 2.09 - 2.07 (m, 4 H), 0.91 (d, *J* = 6.48 Hz, 24 H); δ (for dimeric helicate **2**) 12.85 (s, 8 H), 12.56 (s, 8 H), 12.33 (s, 8 H), 12.08 (s, 8 H), 10.54 (s, 8 H), 10.46 - 10.38 (m, 24 H), 10.24 (s, 8 H), 9.58 (d, *J* = 5.76 Hz, 8 H), 9.30 (d, *J* = 5.40 Hz, 8 H), 9.14 (d, *J* = 5.80 Hz, 8 H), 9.11 (s, 16 H), 8.97 (s, 8H), 8.81 (s, 8H), 8.74 (d, *J* = 6.88 Hz, 8 H), 8.69 - 8.54 (m, 72 H), 8.50 - 8.43 (m, 24 H), 8.11 (s, 16 H), 7.98 - 7.89 (m, 16 H), 7.85 (s, 8 H), 7.78 (t, *J* = 6.64 Hz, 8 H), 7.58 (s, 8 H), 6.17 (t, *J* = 6.28 Hz, 8 H), 4.19 (s, 24 H), 4.13 (s, 24 H), 4.02 (s, 24 H), 3.98 (d, *J* = 5.92 Hz, 32 H), 3.92 (s, 24 H), 3.91 (s, 24 H), 3.31 (s, 24 H), 3.30 (s, 24 H), 2.29 (s, 24 H), 2.11 - 2.09 (m, 16 H), 0.95 - 0.90 (m, 96 H). ^{13}C NMR (CD_3CN, 100 MHz): δ (for dimeric helicate **2**) 177.0, 173.0, 162.4, 162.3, 162.0, 161.4, 161.0, 160.1, 158.9, 155.9, 153.8, 153.1, 151.9, 146.6 146.2, 144.1, 143.5, 143.2, 142.9, 141.7, 139.3, 139.0, 138.8, 137.5, 135.0, 134.7, 133.8, 129.2, 128.1, 127.6, 126.2, 125.1, 123.8, 123.4, 123.2, 122.7, 122.6, 122.5, 105.6, 105.4, 104.9, 104.5, 103.0, 65.9, 57.4, 57.2, 56.9, 56.4, 56.2, 55.8, 30.7, 19.3, 19.2. ESI-MS: *m/z* 1099.6349 $[\mathbf{1}+BF_4]^{3+}$, 1692.4148 $[\mathbf{1}+2(BF_4)]^{2+}$, 1099.6810 $[\mathbf{2}+2(BF_4)]^{6+}$, 1336.7923 $[\mathbf{2}+3(BF_4)]^{5+}$, 1692.4940 $[\mathbf{2}+4(BF_4)]^{4+}$, 2285.9906 $[\mathbf{2}+5(BF_4)]^{3+}$.

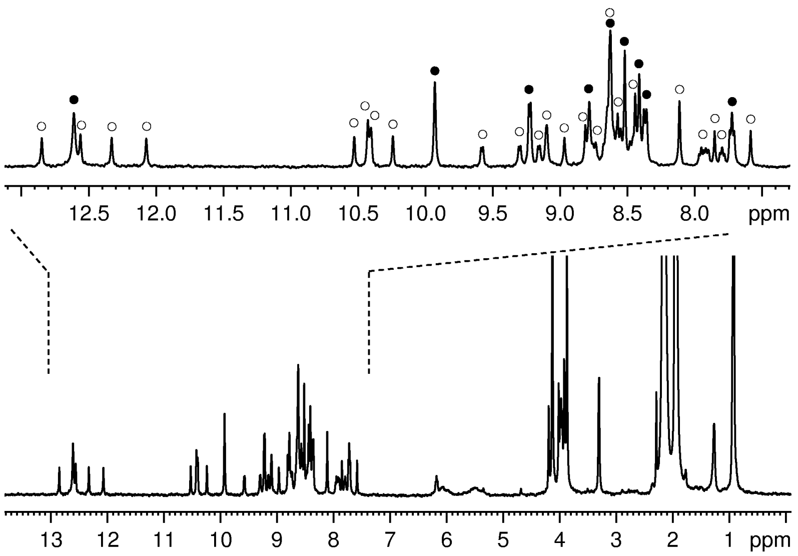

Supplementary Figure 1. ^{1}H NMR spectrum (CD_3CN, 400 MHz, 298 K) of a mixture of monomeric helicate **1** and dimeric helicate **2** when preparing the sample at 1.60 mM concentration of ligand **L**. Signals of the monomeric helicate **1** and of the dimeric helicate **2** are marked with black and empty white circles, respectively.

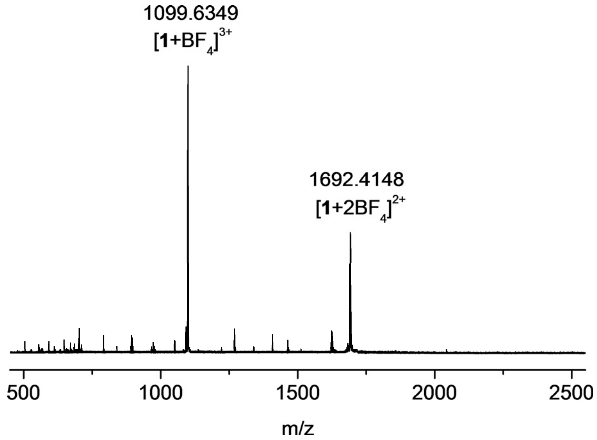

Supplementary Figure 2. Traces of the high-resolution ESI-MS of **1** corresponding to 2+ and 3+ signals. The sample was prepared with low concentration (0.20 mM for ligand concentration). The Mass spectra were recorded immediately after dilution of the sample to 0.02 mM.

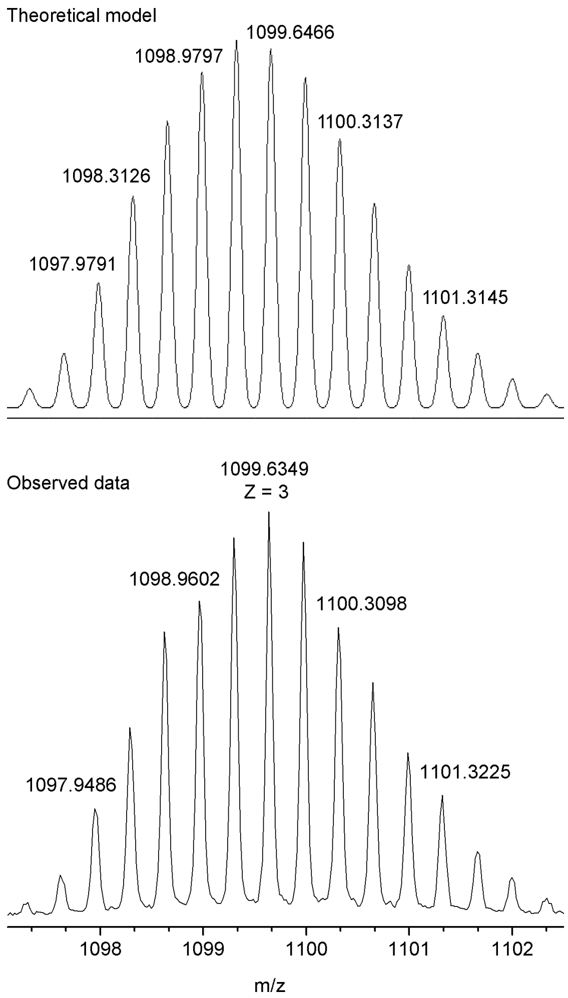

Supplementary Figure 3. High-resolution ESI-MS data for the +3 peaks of **1**.

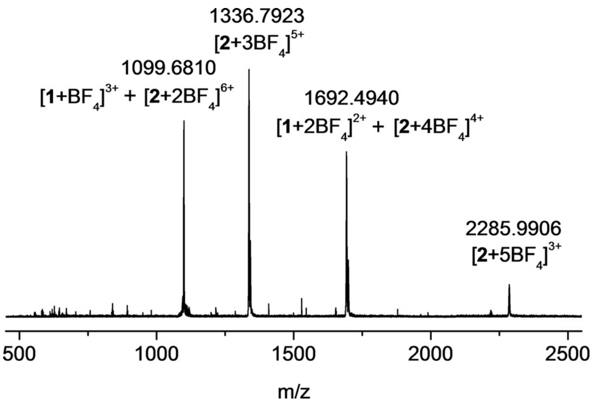

Supplementary Figure 4. Traces of the high-resolution ESI-MS of **2** corresponding to a range of 6+ to 3+ signals. The sample was prepared with high concentration (6.00 mM for ligand concentration). The Mass spectra were recorded immediately after dilution of the sample to 0.02 mM.

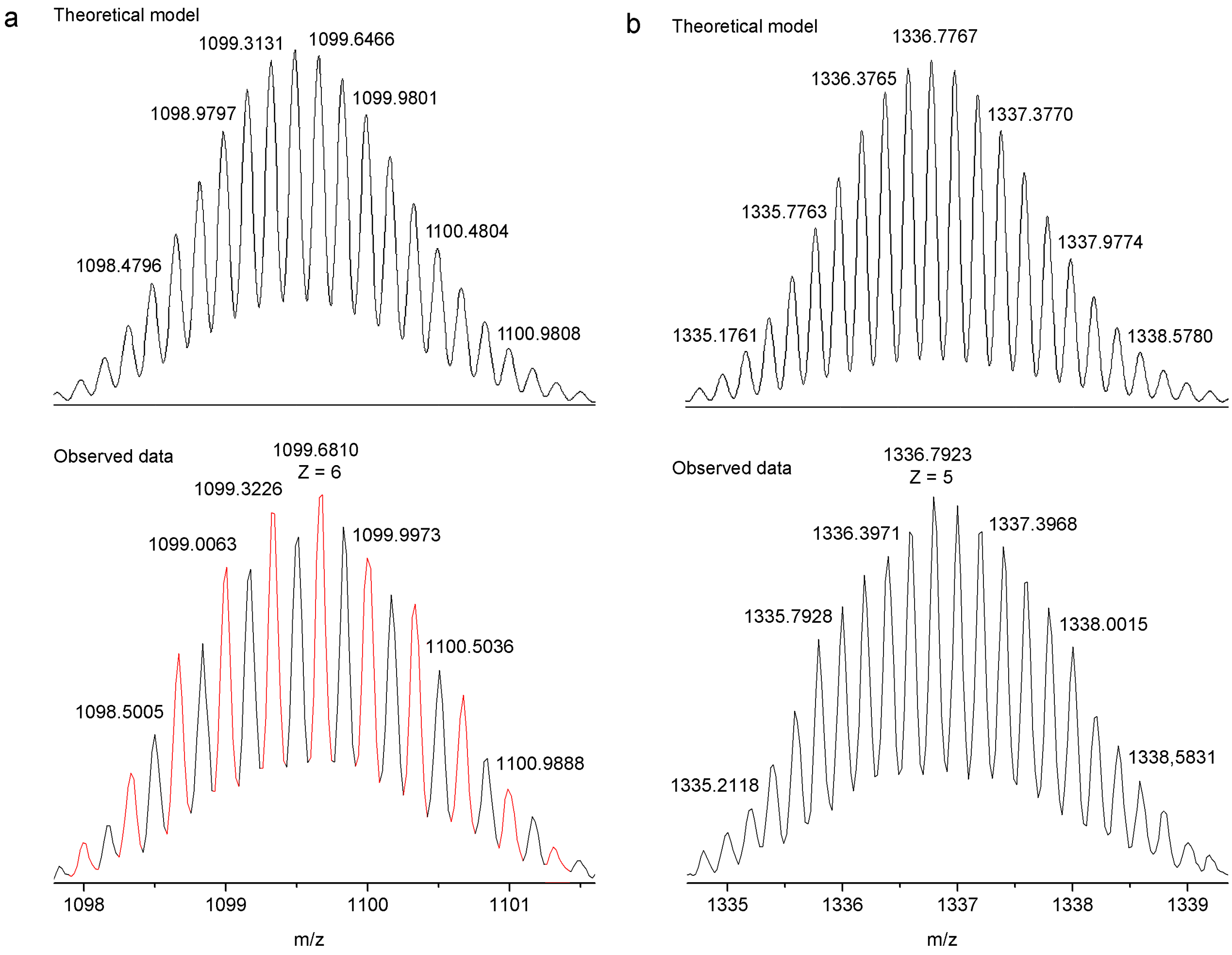

Supplementary Figure 5. (a) High-resolution ESI-MS data for the peaks at 1099.6810. Black denotes the +6 peaks of **2**, and red denote the overlapping parts of the +3 peaks of **1** and the +6 peaks of **2**; (b) High-resolution ESI-MS data for the +5 peaks of **2.**

2.2 Dynamic exchange between the monomeric helicate **1** and dimeric helicate **2**

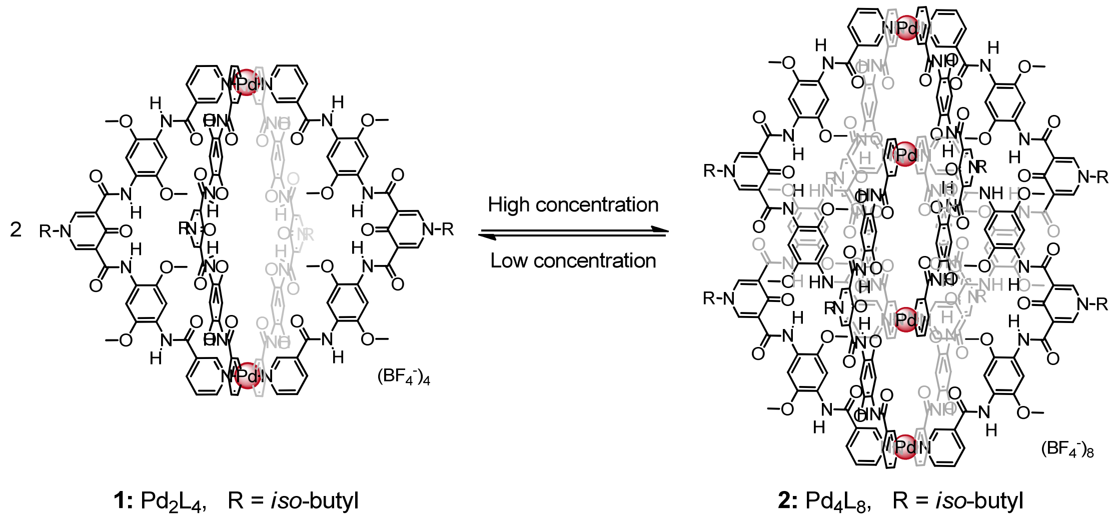

Supplementary Figure 6. Schematic diagram of mutual transformation between the monomeric helicate **1** and dimeric helicate **2**.

For the equilibrium shown in Eq. 1, the equilibrium constant *Ka* of the dimer is given by Eq. 2.

$$2\,[M] \rightleftharpoons [D] \qquad (1)$$

$$Ka = \frac{[D]}{[M]^2} \qquad (2)$$

where: [M] = monomeric helicate **1** concentration; [D] = dimeric helicate **2** concentration

Alternatively,

$$Ka = \frac{n_D \times V_T}{n_M^2} \qquad (3)$$

where: V_T = total volume of the sample; n_M = number of moles of the monomeric helicate; n_D = number of moles of the dimeric helicate

From mass balance,

$$n_M + 2n_D = n_{M0} \qquad (4)$$

where: n_{M0} = initial number of moles of the monomeric helicate

From integration of the NMR spectrum it is possible to obtain the relative ratio of n_M to n_D molar number, a (Eq. 5).

$$n_M / (n_D / 2) = a \qquad (5)$$

Substituting equations (4) and (5) into (3),

$$Ka = \frac{2(a+4) \times V_T}{a^2 \times n_{M0}} \qquad (6)$$

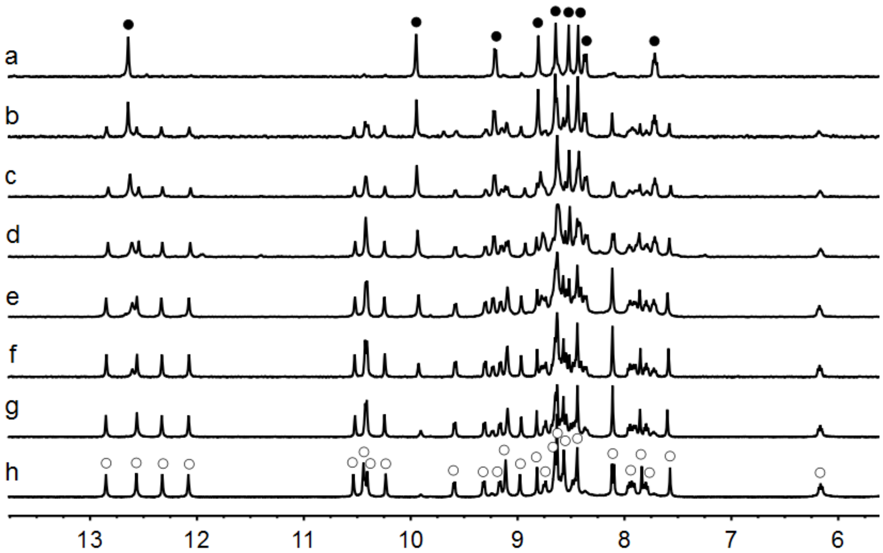

Supplementary Figure 7. Representative ^{1}H NMR spectrum (CD_3CN, 400 MHz, 298 K) of equilibrium of the monomeric helicate **1** and dimeric helicate **2** when preparing the sample at different concentrations of ligand **L**: (a) 0.25 mM, (b) 0.50 mM, (c) 1.50 mM, (d) 2.00 mM, (e) 2.50 mM, (f) 3.50 mM, (g) 5.50 mM, and (h) 8.00 mM. Signals of the monomeric helicate **1** and of the dimeric helicate **2** are marked with black and empty white circles, respectively. *K*a = 1.65 × 10^4 L mol^{-1}.

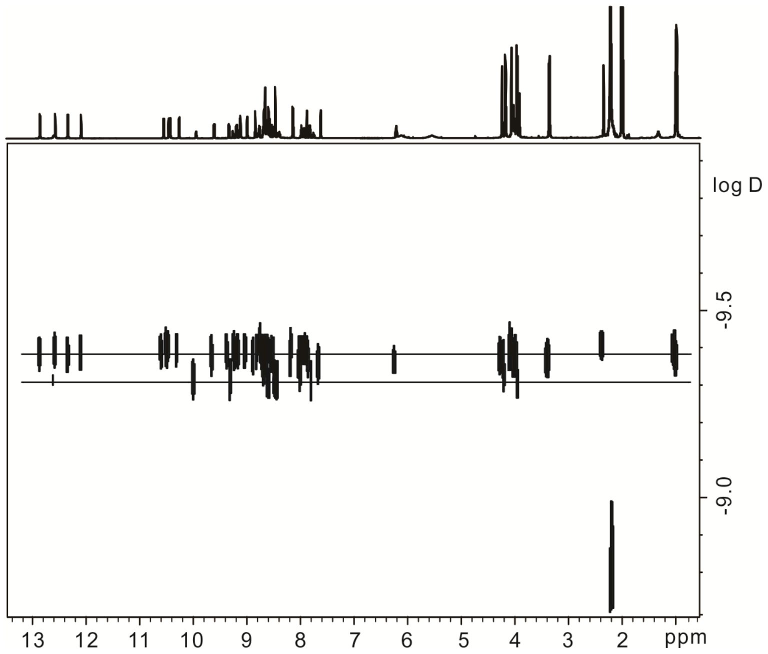

Supplementary Figure 8. ^{1}H DOSY NMR spectrum of a mixture of monomeric helicate **1** and dimeric helicate **2** in CD_3CN at 298 K. The diffusion coefficient for **1** and **2** in this solvent system was measured to be 4.90×10^{-10} and 4.15×10^{-10} m^2 s^{-1}, respectively.

Hydrodynamic radii were calculated from the diffusion values using the Stokes-Einstein equation:

$$r = \frac{K_B \cdot T}{6 \cdot \pi \cdot \eta \cdot D}$$

where: r = radius; K_B = Boltzmann's constant (1.38×10^{-23} $J{\cdot}K^{-1}$); T = temperature (298 K), η = dynamic viscosity of CD_3CN (3.69×10^{-4} $Pa{\cdot}s^{-1}$); and D = diffusion values estimated by the DOSY experiment. The hydrodynamic radii of single and double helicates were calculated to be 1.2 nm and 1.4 nm, respectively.

2.3 Full characterization of the dimeric helicate **2**

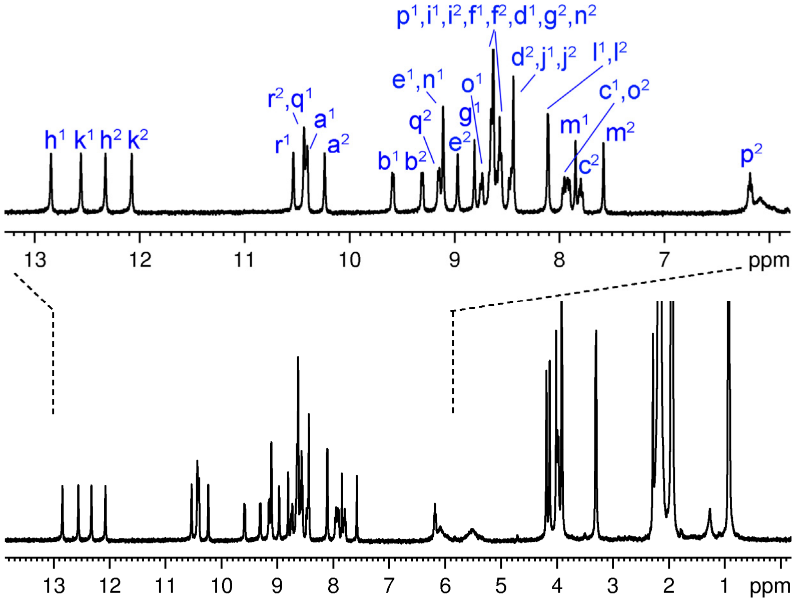

Supplementary Figure 9. ^{1}H NMR spectrum (CD_3CN, 400 MHz, 298 K) of **2** with assignment of signals when preparing the sample at 8.40 mM concentration of ligand **L**. The numbers of different isomers were assigned at superscript.

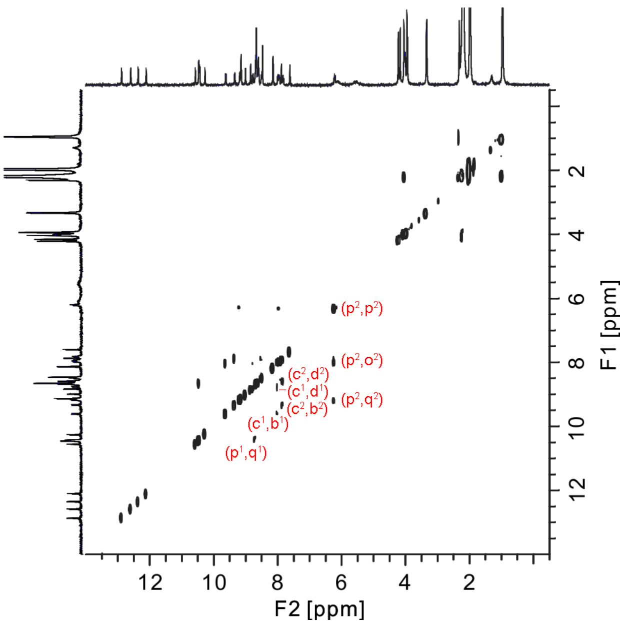

Supplementary Figure 10. ^{1}H-^{1}H COSY spectra (CD_3CN, 400 MHz, 298 K) of **2**.

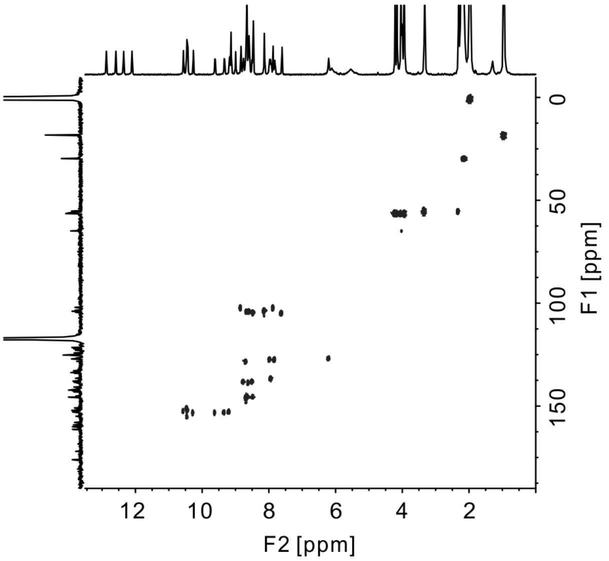

Supplementary Figure 11. HSQC spectra (CD_3CN, 400 MHz, 298 K) of **2**.

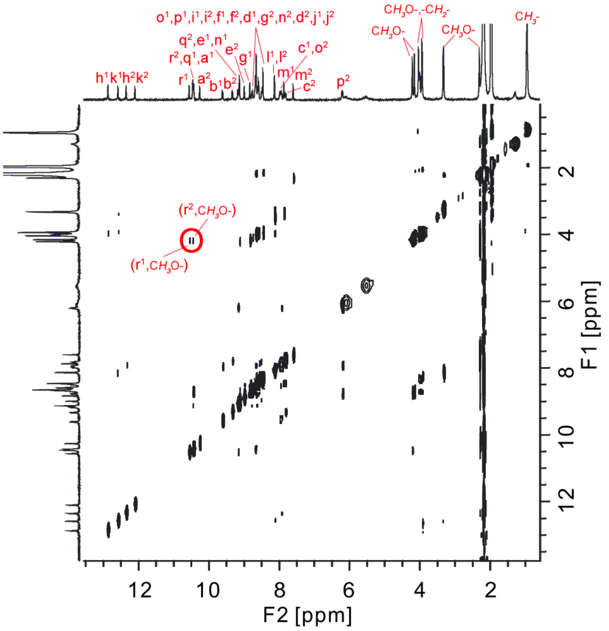

Supplementary Figure 12. ^1H-^1H NOESY spectra (CD_3CN, 400 MHz, 298 K) of **2**. The observation of the cross peaks between the signals of methoxyl (-OCH_3) and pyridine protons (r) shed light on its interlocked architecture of **2**. Also see the schematic representation of modelling structures of **2** in Supplementary Figure 18.

3 Method for definition of P/M helicity

The azimuthal angle θ is commonly used to describe the helicity for M_2L_4 helicates.[5] A structure is qualified to be a helicate with helicity when the azimuthal angle θ is non zero. This method is based on the torsion angle of coordinating segments with respect to the Pd-Pd axis (i.e., $N_{pyridine}$-Pd-Pd-$N_{pyridine}$ angle), not just in view of the amide torsion angle.

Although it is convenient to use this parameter to describe the helicity for M_2L_4 helicates, it has some drawbacks. In our case, the ligands can be subjected to a distortion or twisting in the center and partitioned into two sections, each of which can thus coordinate the metal ion to different chiralities (*P* and *M*), leading to a meso complex. The azimuthal angle is equal to zero, but the ligands still show the helical conformation.

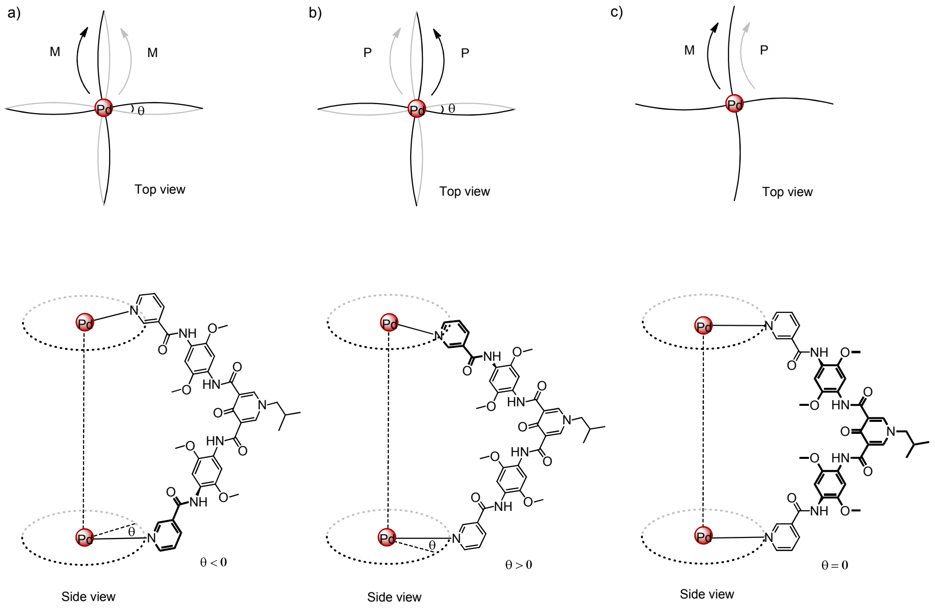

Supplementary Figure 13. Schematic diagrams for the definition of azimuthal angle θ of monomeric helicates. Only one ligand per single helicate is depicted for clarity. a) *MM* monomeric helicate with a definite azimuthal angle ($\theta < 0$), b) *PP* monomeric helicate ($\theta > 0$), c) *MP* monomeric helicate ($\theta = 0$).

In order to better assign the helicity in our system, it is necessary to horizontally divide a monomeric helicate into two equal parts and quantify the helicity in a piecewise manner. Therefore, to characterize the helicity, it needs two symbols (XX, X = *P* or *M*) for a monomeric helicate, and four symbols (XXXX, X = *P* or *M*) for a dimeric helicate, given the consistency of twisting of ligands in the individual helicate. The approach to assign the helicity is presented as follow:

1) Determine the azimuthal angle.
2) If the azimuthal angle is not zero, observe the angle phase. When it is clockwise spin from the top-to-down view, the corresponding segment is defined with *M* helicity, and assigned to *P* helicity

when angle phase is anticlockwise.

3) If the azimuthal angle is zero, observe the rotation direction of the twisted ligand around the metal. When it is clockwise from the top-to-down view, the corresponding segment is defined with *M* helicity, and assigned to *P* helicity when the direction is anticlockwise.

This method for *P*/*M* helicity definition is consistent with the description in the main text where helicity is defined by the helical orientation of each Pd^{2+} cation with respect to the center of the ligand.

4 Solution spectroscopic analysis of the helicate 1-Pt

4.1 Self-assembly of the monomeric helicate **1-Pt**

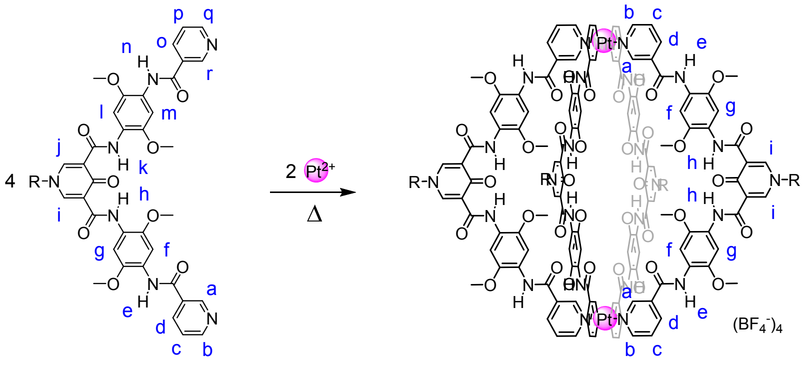

To a NMR tube were added ligand **L** (0.42 mg, 0.56 μmol), $Pt(CH_3CN)_4(BF_4)_2$ (0.15 mg, 0.28 μmol)[4] and CD_3CN (500 μL). The reaction mixture was placed at 80 °C for one day to give a 0.28 mM solution of monomeric helicate **1-Pt**. ^{1}H NMR (CD_3CN, 400 MHz): δ 12.65 (s, 8 H), 12.60 (s, 8 H), 9.87 (s, 8 H), 9.19 (d, *J* = 5.44 Hz, 8 H), 9.13 (s, 8 H), 8.93 (s, 8 H), 8.91 (d, *J* = 7.36 Hz, 8 H), 8.84 - 8.82 (m, 16 H), 8.70 (s, 8 H), 8.65 (s, 8 H), 8.55 (s, 8 H), 8.53 (s, 8 H), 8.44 (s, 8 H), 8.37 (d, *J* = 8.24 Hz, 8 H), 8.13 - 8.09 (m, 16 H), 7.71 (t, *J* = 6.96 Hz, 8 H), 4.13 (s, 24 H), 4.03 - 4.01 (m, 32 H), 3.98 (d, *J* = 7.12 Hz, 24 H), 3.92 (s, 24 H), 3.88 (s, 24 H), 2.07 - 2.06 (m, 8 H), 0.97 - 0.88 (m, 96 H). ESI-MS: *m/z* 1158.3643 $[\mathbf{1\text{-}Pt}+BF_4]^{3+}$, 1781.0426 $[\mathbf{1\text{-}Pt}+2BF_4]^{2+}$.

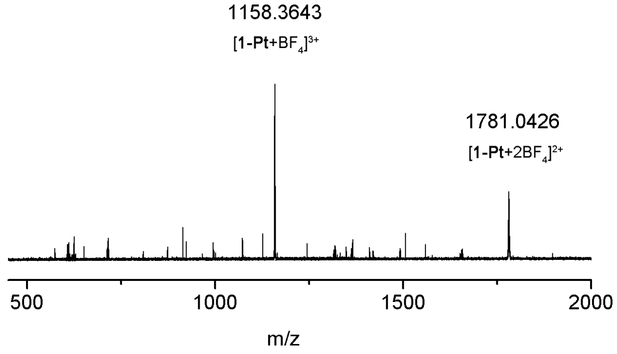

Supplementary Figure 14. Traces of the high-resolution ESI-MS of **1-Pt** corresponding to 2+ and 3+ signals.

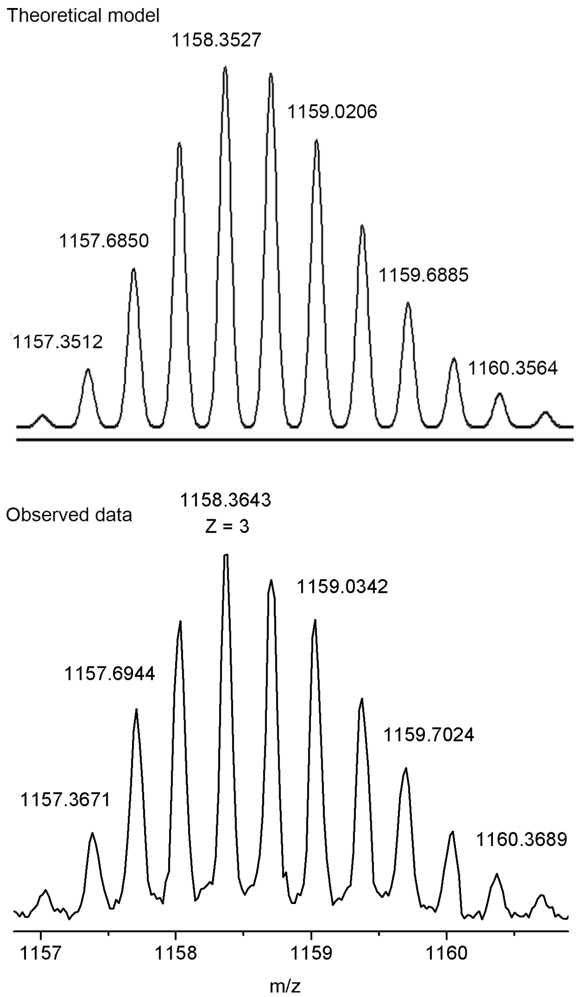

Supplementary Figure 15. High-resolution ESI-MS data for the +3 peaks of **1-Pt**.

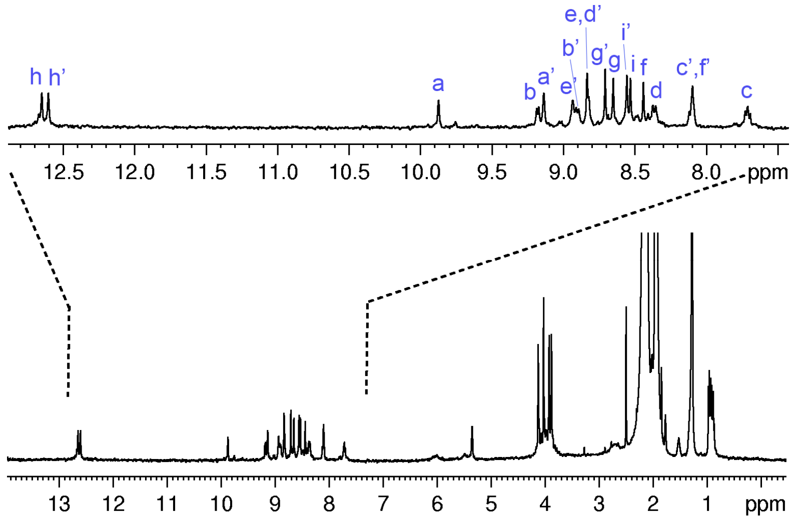

Supplementary Figure 16. ^{1}H NMR spectrum (CD_3CN, 400 MHz, 298 K) of **1-Pt** with assignment of signals.

4.2 The *P*/*M* helical interconversion of helicate **1-Pt** induced by anion guests

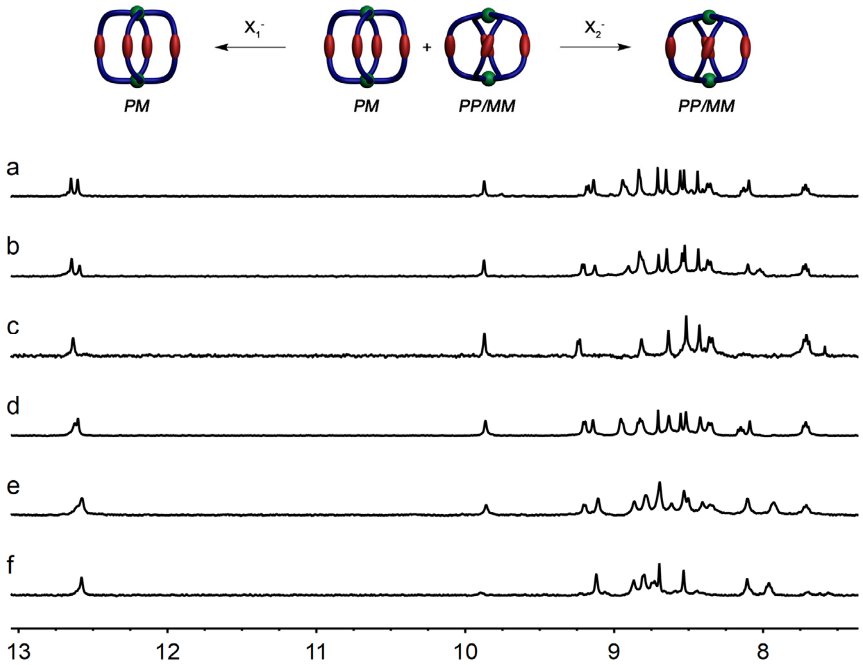

Supplementary Figure 17. Representative ^{1}H NMR spectrum (CD_3CN, 400 MHz) of **1-Pt** (0.28 mM): a) monomeric helicate **1-Pt** (the mixture of PP/MM and PM); addition of b) 0.50 equiv. and c) 1.0 equiv. SO_4^{2-} to the solution of **1-Pt**; addition d) 0.50 equiv., e) 1.0 equiv. and f) 2.0 equiv. Cl^- to the solution of **1-Pt**. The signs of X_1^- and X_2^- represent Cl^- or SO_4^{2-} anion.

5 Molecular Modelling of 2

The amide twisting allows **2** to have 10 possible isomers (four pairs of enantiomers including *PPPP/MMMM*, *PPPM/MMMP*, *PPMP/MMPM*, and *PMMP/MPPM*, two meso compounds *PPMM*, and *PMPM*). Whereas there are just four sets of signals in ^{1}H NMR for **2**, which means two kinds of diastereomers are thermodynamic stable. To find out which isomers are more stable, the simulations for all isomers were carried out by DFT (B3LYP/LANL2DZ) calculation.

Firstly, three possible isomers (*PP*, *MM*, and *PM*) of monomeric helicate **1** were built using the software Spartan'14[6] and roughly optimized by molecular mechanics methods (MMFF). Subsequently, the structural refinement of these three isomers were performed using the software Gauss'09[7] on the B3LYP/LANL2DZ level of theory (Charge: +4, Spin multiplicity: 1, no constraints). Whereafter, we combined those isomers of **1** to get 10 isomers of **2**. As some of those isomers are enantiomers, we just used 6 isomers for calculation.

These structures were further subjected to a DFT calculation by using the seam level of 1. Constraints were used to place the Pd^{II}-cations on the central axis to avoid their deviation. Although there are too many numbers of atoms in **2**, these structures were optimized until the Force is converged. These structures obtained from DFT calculations as well as energies are summarized in Supplementary Table 1.

Supplementary Table 1: Geometry optimization for six possible isomers of **2**.

	PPPP	*PPPM*
Structure	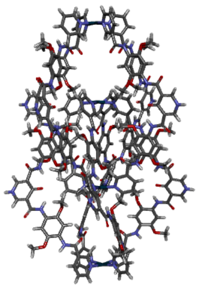 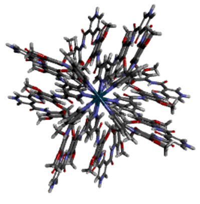	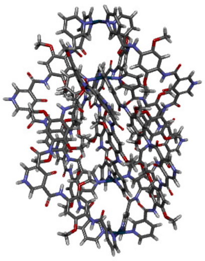 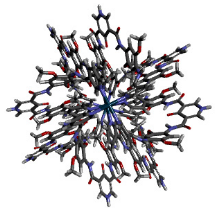
Energy (kcal/mol)	-12,429,369.902813448	-12,429,361.544945007
	PMMP	*PPMP*

Structure	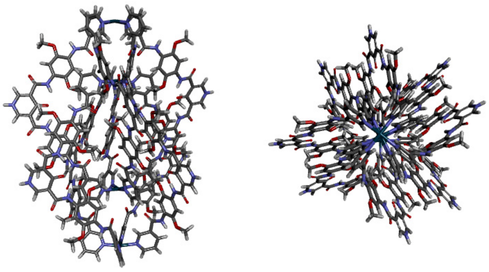	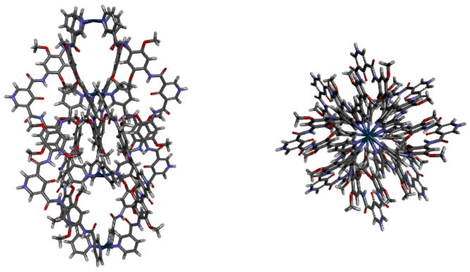
Energy (kcal/mol)	-12,429,369.005160393	-12,429,358.53120273
	PPMM	*PMPM*
Structure	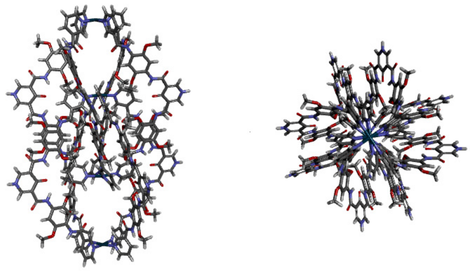	**Unfortunately, the calculation of *PMPM* was failed without SCF convergence.**
Energy (kcal/mol)	-12,429,355.614159744	NONE

The models revealed that the isomers (*PPPP*, *PMMP*) are more stable than other isomers. It seems the helicate dimerization prefer occurring between two monomeric helicates with the same helicity rather than with the different helicity. Moreover, the tight packing of the aromatic rings could be observed when the dimeric helicates adopted the same ligand twisting at the intertwined section. These modelling results are consistent with the NMR experiments, which showed equally two sets of signals for **2**. Furthermore, the energy modelling correlates best with the X-ray crystallographically determined structures of the dimeric helicate [2Cl⊂**2**] (only showing *PPPP*/*MMMM* helicity) with encapsulation of two chloride anions (see below).

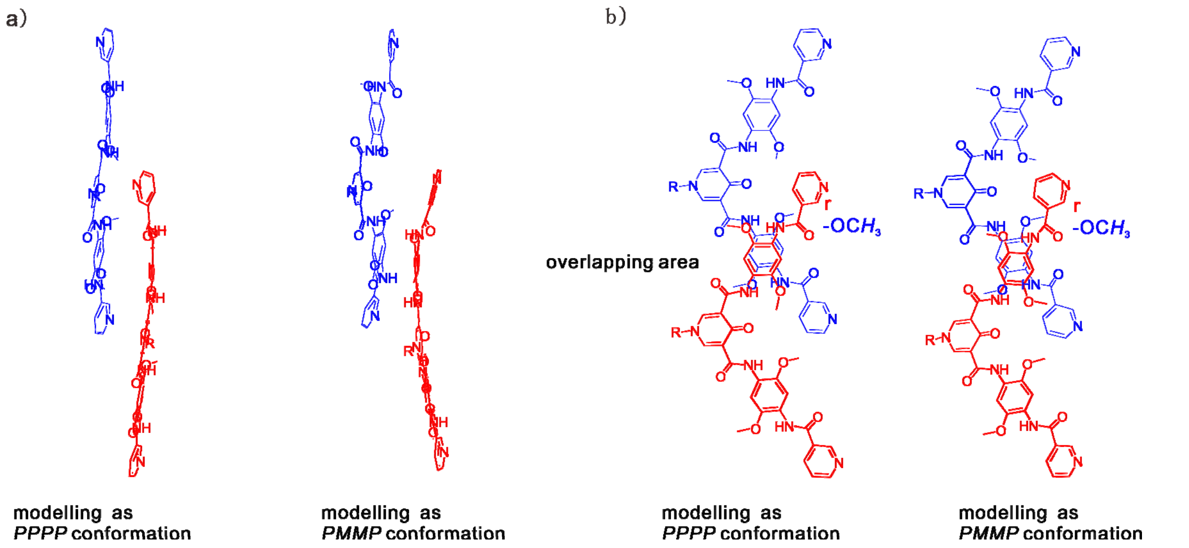

Supplementary Figure 18. Schematic representation of modelling structures (*PPPP* and *PMMP*) of sectional **2** showing the overlapping area of the ligands. a) side view, b) front view.

6 Solution spectroscopic analysis of the chloride complex [2Cl⊂**2**]

6.1 Self-assembly of the complex [2Cl⊂**2**]

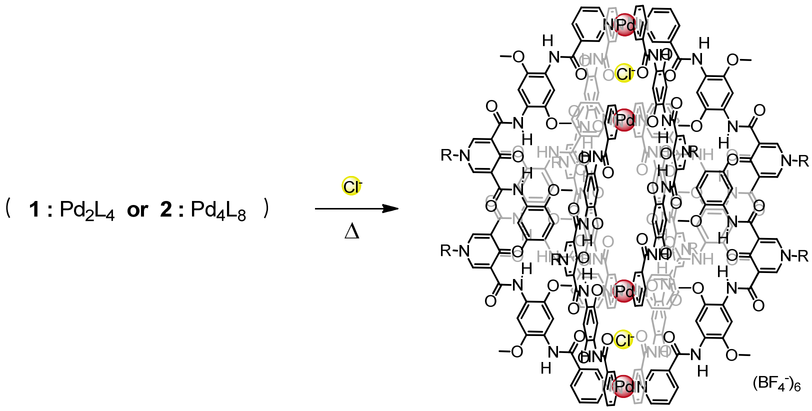

[2Cl⊂**2**]: [2Cl+(Pd_4L_8)], R = *iso*-butyl

The complex [2Cl⊂**2**] were formed by the mixture solution of monomeric helicate **1** and dimeric helicate **2** which obtained above with NBu_4Cl (0.70 μmol, 40 μL of a 17.5 mM stock solution in CD_3CN) at 56 °C for 12 h in a NMR tube to give a 0.32 mM solution of the complex [2Cl⊂**2**]. ^{1}H NMR (CD_3CN, 400 MHz): δ 12.35 (s, 8 H), 11.94 (s, 16 H), 11.41 (d, J = 5.44 Hz, 8 H), 10.46 (s, 8 H), 9.50 (d, J = 5.56 Hz, 8 H), 8.76 (s, 8 H), 8.72 (s, 8 H), 8.56 (s, 8 H), 8.48 (s, 8 H), 8.43 - 8.40 (m, 16 H), 8.24 (s, 8 H), 8.12 - 8.08 (m, 16 H), 7.83 (t, J = 6.96 Hz, 8 H), 7.50 (d, J = 6.84 Hz, 8 H), 7.22 (s, 8 H), 3.94 - 3.91 (m, 16 H), 3.87 (s, 24 H), 3.78 (s, 24 H), 3.40 (s, 24 H), 2.56 (s, 24 H), 2.09 - 2.08 (m, 8 H), 0.93 - 0.91 (m, 48 H). ESI-MS: m/z 1082.4785 [2Cl⊂**2**]$^{6+}$, 1316.1803 [(2Cl⊂**2**)+BF_4]$^{5+}$, 1666.9798 [(2Cl⊂**2**)+2(BF_4)]$^{4+}$.

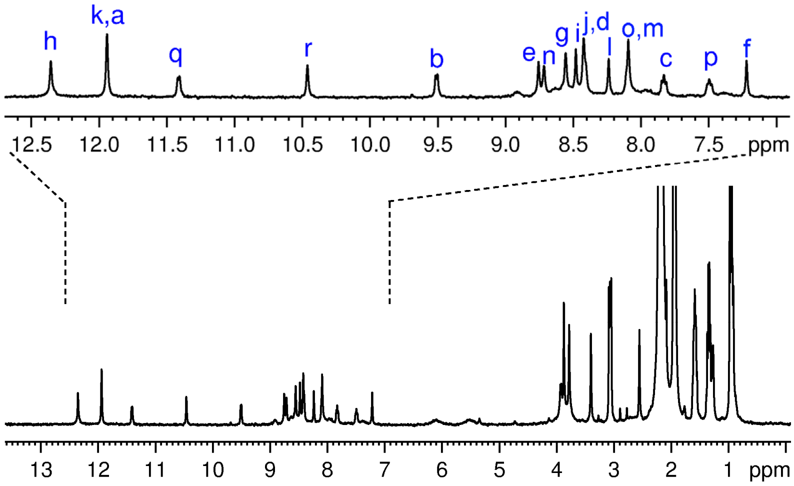

Supplementary Figure 19. ^{1}H NMR spectrum (CD_3CN, 400 MHz, 298 K) of [2Cl⊂**2**] with assignment of signals.

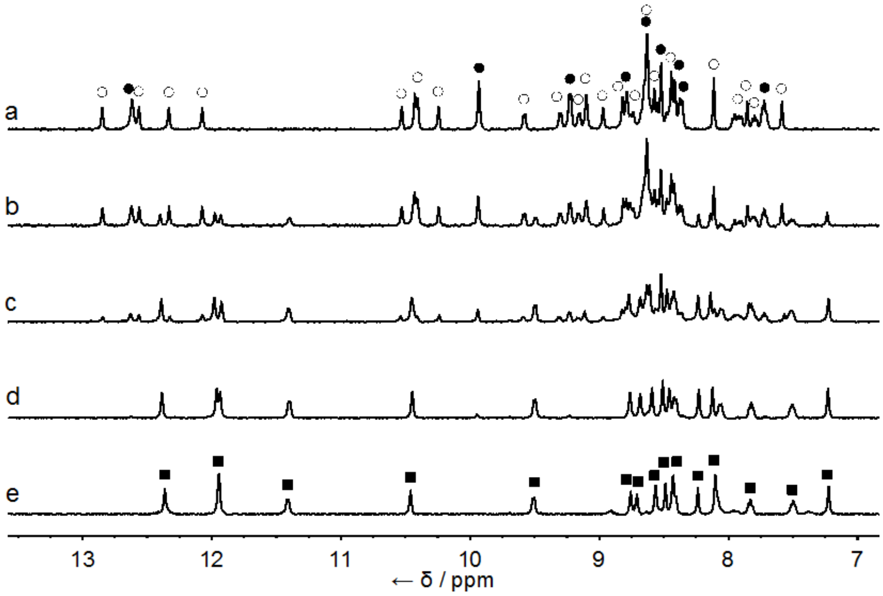

Supplementary Figure 20. Representative ^{1}H NMR spectrum (CD_3CN, 400 MHz, 298 K) of the mixture of monomeric helicate **1** and dimeric helicate **2** (ca. [**1**]/2 + [**2**] = 0.2 mM) with addition of *n*BuN^+Cl^-: (a) 0 mM, (b) 0.15 mM, (c) 0.30 mM, (d) 0.45 mM, (e) 0.6 mM at equilibrium. The formation of [2Cl⊂**2**] is saturated after addition more than 3 equiv. of Cl^- to the solution of **2**. Addition over 5 equiv. of Cl^- anions leads to the decrease of [2Cl⊂**2**] signal intensities accompanied by precipitation of the complex. Signals of the monomeric helicate **1** and the dimeric helicate **2** are marked with black and empty white circles, respectively. And signals of the dimeric helicate [2Cl⊂**2**] are marked with black squares.

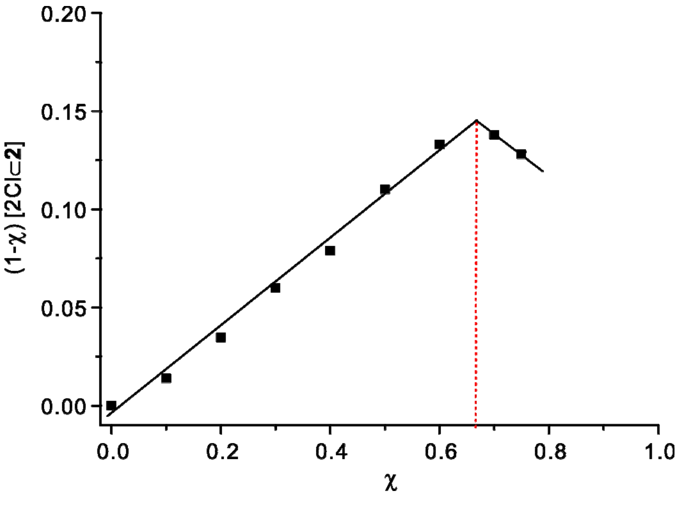

Supplementary Figure 21. Job's plot of helicate with *n*BuN^+Cl^-. Maintain a total concentration equal to [**1**]/2 + [**2**] + [Cl^-] = 0.5 mM, and define the ratio of Cl^-/total concentration as a molar fraction χ = [Cl^-]/([**1**]/2 + [**2**] + [Cl^-]). The inflexion point at 0.66 shows a 1:2 (**2**/Cl^-) stoichiometry. The plot is missing at the range of high ratio of Cl^- due to the precipitation of the complex.

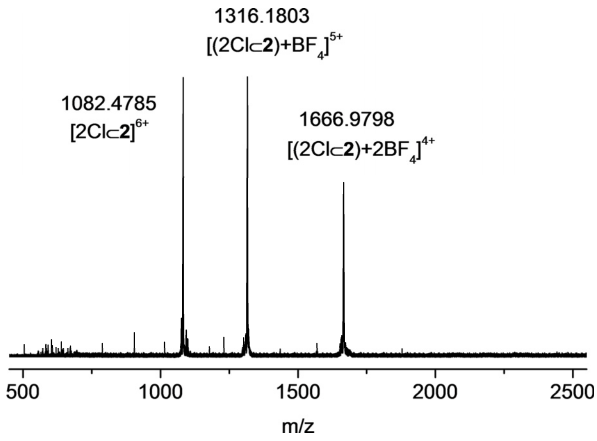

Supplementary Figure 22. Traces of the high-resolution ESI-MS of [2Cl⊂**2**] corresponding to 4+, 5+ and 6+ signals.

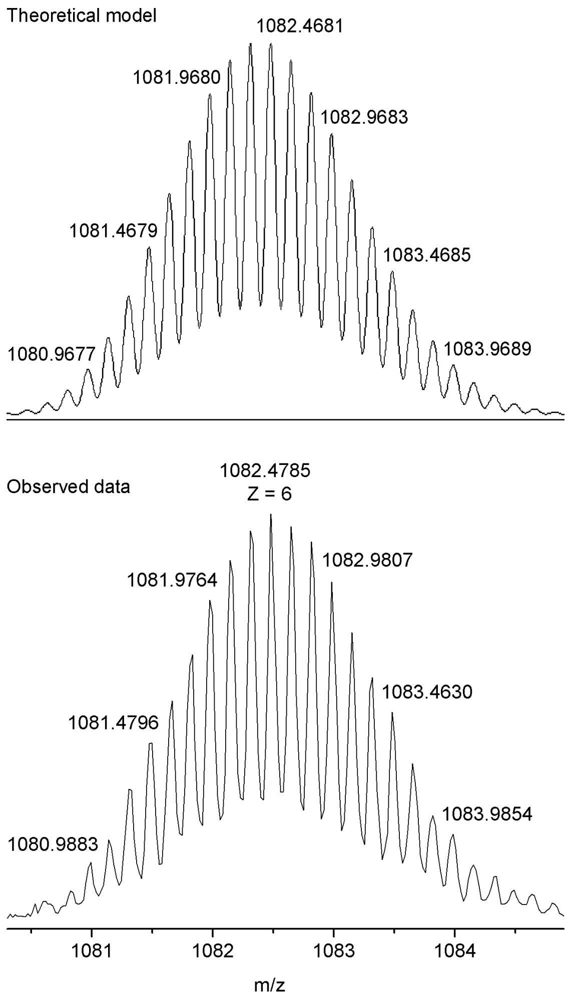

Supplementary Figure 23. High-resolution ESI-MS data for the +6 peaks of [2Cl⊂**2**].

6.2 Capturing and releasing chloride anions within the dimeric helicate

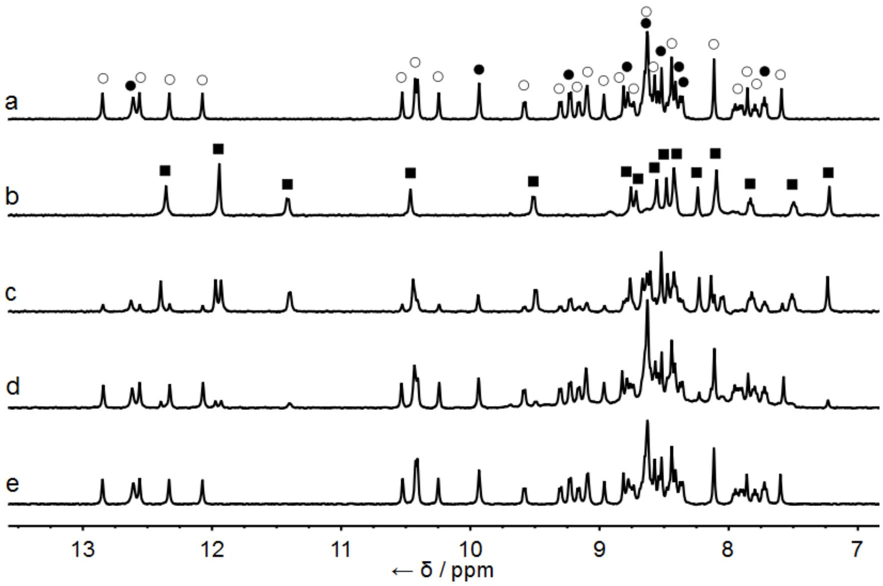

Supplementary Figure 24. Representative ^{1}H NMR spectrum (CD_3CN, 400 MHz, 298 K) of (a) the mixture of monomeric helicate **1** and dimeric helicate **2** (ca. [**1**] + 2[**2**] = 0.60 mM), (b) dimeric helicate [2Cl⊂**2**] before the addition of $AgBF_4$, and (c) one day, (d) seven days, (e) twelve days after addition of $AgBF_4$ at room temperature. Signals of the monomeric helicate **1** and of the dimeric helicate **2** are marked with black and empty white circles, respectively. And signals of the dimeric helicate [2Cl⊂**2**] are marked with black squares.

7 X-Ray Crystallography

Single crystal [2Cl⊂**2**] was grown by slow diffusion of n-butyl ether in the acetonitrile solutions of the self assemblies. A single crystal of [2Cl⊂**2**] in mother liquor was pipetted onto a glass containing Paratone-N oil. To avoid collapse of the crystal lattice, the crystal was quickly mounted onto a nylon loop and immediately flash cooled in liquid nitrogen.

Crystallographic data were all collected at the the Analysis and Testing Center in Huazhong University of Science and Technology (HUST) on a Rigaku MM007 HF rotating anode (0.8 kW). Data were diffracted at the CuK_α wavelength, and data-collection strategies were based on Omega scans at 100(2) K. The Rigaku CrystalClear suite versions 2.0 were used to index, integrate and scale the data with a multi-scan absorption correction.

The structures were solved by direct methods using SHELXT[8] and refined against F^2 on all data by full-matrix least squares with SHELXL[9] following established refinement strategies.[10] Most of the non-H atoms were refined with anisotropic temperature parameters, the disordered ones were refined with isotropic temperature parameters. All hydrogen atoms, were included into the model at geometrically calculated positions and refined using a riding model. SHELX ISOR and DELU restraints were used in the refinement strategy in order to reduce the anisotropic displacement parameters of the side chains. DFIX instructions were used to geometrically restraint most of the side chains. The contribution of the electron density associated with disordered solvent molecules, which could not be modelled with discrete atomic positions were handled using the SQUEEZE[11] routine in PLATON.[12,13] Crystallographic data have been deposited with the CCDC, under deposition number CCDC 1893706.

Supplementary Table 2: Crystal data and structure refinement for the interpenetrated helicate.

Formula	C316 H320.58 B4 Cl2 F16 N58 O72 Pd4
M	6926.61
Crystal system	monoclinic
Space group	C2/c
a/Å	25.0095(5)
b/Å	46.1941(4)
c/Å	41.8030(6)

α/°	90
β/°	104.231(2)
γ/°	90
V/Å^3	46812.6(13)
T /K	100.00(10)
Z	4
ρ/g cm^{-1}	0.983
size (mm)	0.02 × 0.01 × 0.01
λ/ Å	1.54184
μ/mm^{-1}	1.897
Independent reflections	25615
measured reflections	258608
parameters/restraints	2142/95
R1, wR2	0.0861, 0.2677
goodness of fit	1.017

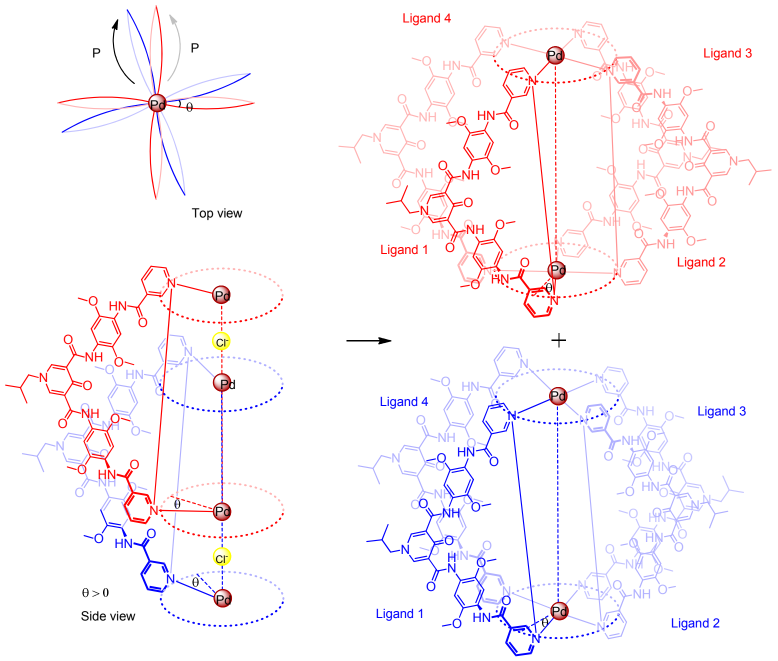

Supplementary Figure 25. Schematic diagrams for determination of helicity for the crystal structure of [2Cl⊂**2**] with *PPPP* conformation. The azimuthal angles for each ligands were measured and summarized in the following Supplementary Table 3. The azimuthal angles all show the anticlockwise spin from the top-to-down view, indicating all the ligands have a coincident helicity (i.e., *P* helicity).

Supplementary Table 3. The azimuthal angle θ of the dimeric helicate [2Cl⊂**2**] from the crystal structure.

θ	ligand 1	ligand 2	ligand 3	ligand 4
upper helicate	27.61	32.53	27.61	32.53
upper helicate	29.83	27.85	29.83	27.85

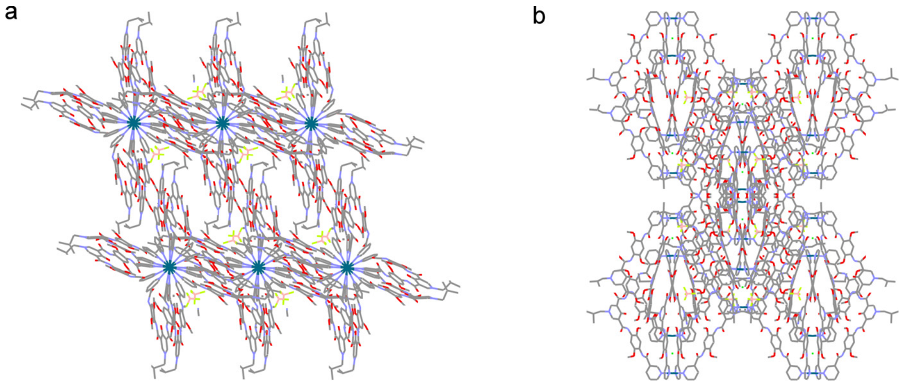

Supplementary Figure 26. Packing of [2Cl⊂**2**] in the unit cell: (a) view along the b-axis, (b) view along the c-axis. Dimeric helicates, anions and solvent were displayed as wireframes.

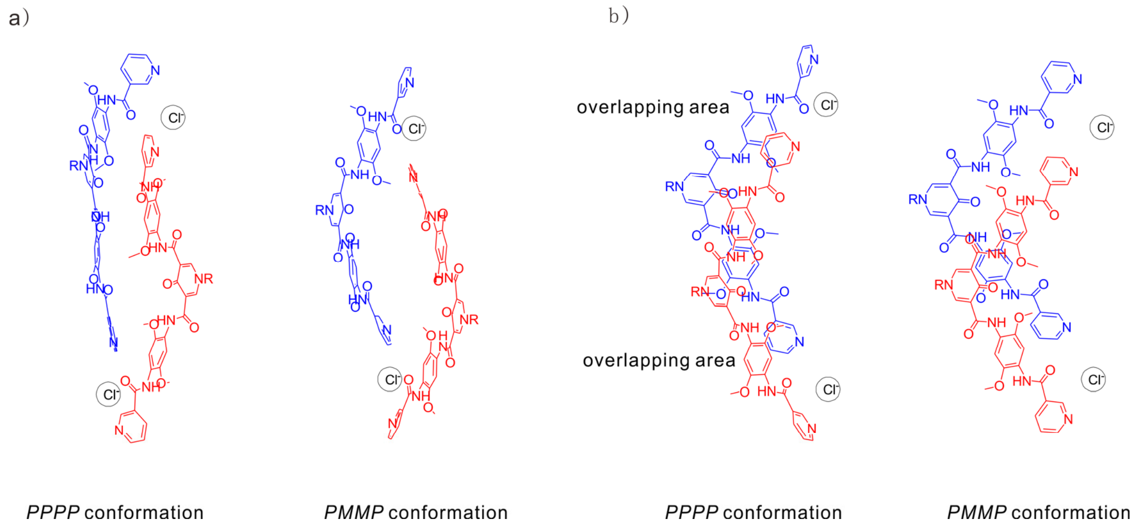

Supplementary Figure 27. a) Side view, and b) front view of structural imitation of sectional [2Cl⊂**2**] with *PPPP* and *PMMP* conformations. The fake structures [2Cl⊂**2**] was fabricated based on the modelling of **2**. The results shown the aromatic stacking of *PPPP* isomer was more effective than that of *PMMP* isomer.

8 Solution spectroscopic analysis of the helicates 3R and 4R

8.1 Self-assembly of the monomeric helicate **3**R and dimeric helicate **4**R (or **3**S/**4**S)

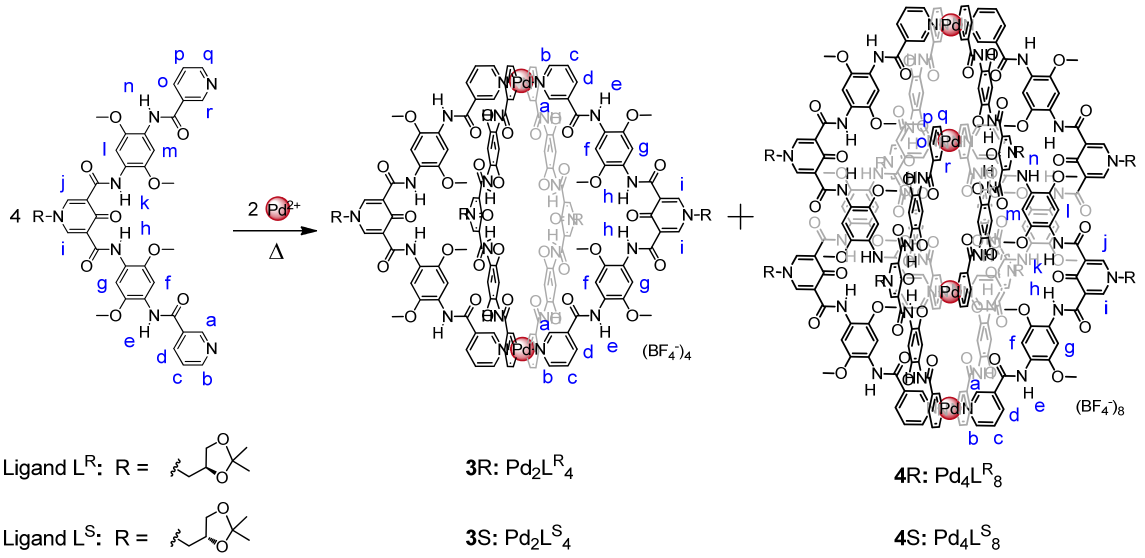

To a NMR tube were added ligand $\mathbf{L}^{R}$ (or $\mathbf{L}^{S}$, 1.13 mg, 1.40 μmol), $Pd(CH_3CN)_4(BF_4)_2$ (0.31 mg, 0.70 μmol) and CD_3CN (500 μL). The reaction mixture was placed at 56 °C for 12 h to give a mixture solution of monomeric helicate **3**R and dimeric helicate **4**R (or **3**S/**4**S). ^{1}H NMR (CD_3CN, 400 MHz): δ (for monomeric helicate **3**S) 12.59 (s, 8 H), 9.95 (s, 8 H), 9.21 (d, *J* = 3.20 Hz, 8 H), 8.79 (s, 8 H), 8.69 (s, 8 H), 8.52 (s, 8 H), 8.43 (s, 8 H), 8.36 (d, *J* = 7.84 Hz, 8 H), 7.70 (t, *J* = 6.40 Hz, 8 H), 4.47 - 4.43 (m, 4 H), 4.38 - 4.34 (m, 4 H), 4.19 - 4.16 (m, 4 H), 4.13 (s, 24 H), 3.96 - 3.91 (m, 4 H), 3.87 (s, 24 H), 3.73 - 3.69 (m, 4 H), 1.37 (s, 12 H), 1.27 (s, 12 H); δ (for dimeric helicate **4**S) 12.82 (s, 8 H), 12.79 (s, 8 H), 12.55 (s, 8 H), 12.52 (s, 8 H), 12.39 (s, 8 H), 12.28 (s, 8 H), 12.06 (s, 8 H), 12.01 (s, 8 H), 10.57 - 10.52 (m, 16 H), 10.46 - 10.43 (m, 32 H), 10.40 (s, 16 H), 10.27 (s, 8 H), 10.21 (s, 8 H), 9.59 (t, *J* = 4.80 Hz, 16 H), 9.31 (t, *J* = 5.96 Hz, 16 H), 9.20 - 9.15 (m, 16 H), 9.14 - 9.10 (m, 32 H), 8.99 (s, 16 H), 8.80 - 8.74 (m, 32 H), 8.71 - 8.69 (m, 32 H), 8.68 - 8.64 (m, 40 H), 8.62 - 8.60 (m, 24 H), 8.59 - 8.57 (m, 32 H), 8.49 (t, *J* = 7.68 Hz, 32 H), 8.43 (d, *J* = 3.32 Hz, 16 H), 8.17 (d, *J* = 3.92 Hz, 16 H), 8.06 (d, *J* = 3.32 Hz, 16 H), 7.95 (t, *J* = 7.04 Hz, 24 H), 7.90 (s, 8 H), 7.86 (d, *J* = 7.16 Hz, 8 H), 7.83 - 7.79 (m, 24 H), 7.61 (s, 8 H), 7.48 (s, 8 H), 6.22 (t, *J* = 6.00 Hz, 8 H), 6.12 (t, *J* = 7.16 Hz, 8 H), 4.74 - 4.70 (m, 5 H), 4.65 - 4.60 (m, 7 H), 4.49 - 4.42 (m, 30 H), 4.38 - 4.32 (m, 32 H), 4.23 - 4.21 (m, 24 H), 4.20 - 4.13 (m, 122 H), 4.11 - 4.09 (m, 32 H), 4.04 - 4.00 (m, 60 H), 3.95 -3.92 (m, 72 H), 3.89 - 3.87 (m, 24 H), 3.76 - 3.70 (m, 40 H), 3.35 - 3.32 (m, 72 H), 3.28 - 3.26 (m, 24 H), 1.52 - 1.50 (m, 24 H), 1.41 - 1.39 (m, 48 H), 1.38 - 1.36 (m, 48 H), 1.29 - 1.26 (m, 72 H). ESI-MS: *m/z* 1176.9855 $[(\mathbf{3}S)+BF_4]^{3+}$, 1429.5992 $[(\mathbf{4}S)+3(BF_4)]^{5+}$.

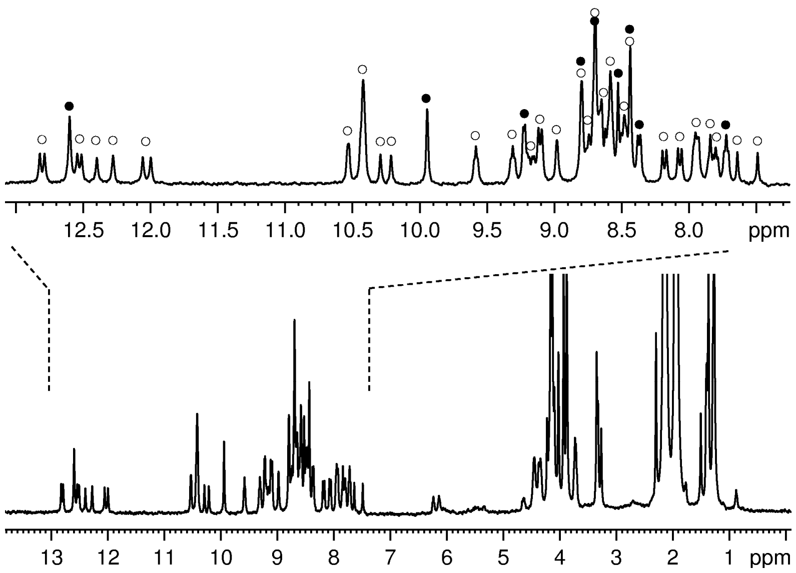

Supplementary Figure 28. ^{1}H NMR spectrum (CD_3CN, 400 MHz, 298 K) of a mixture of monomeric helicate **3**S and dimeric helicate **4**S when preparing the sample at 1.60 mM concentration of ligand $\mathbf{L}^S$. Signals of the monomeric helicate **3**S and of the dimeric helicate **4**S are marked with black and empty white circles, respectively.

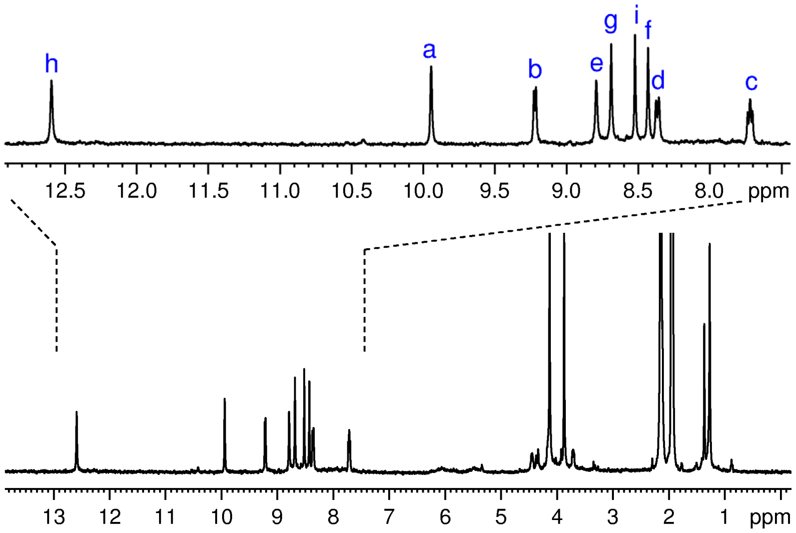

Supplementary Figure 29. ^{1}H NMR spectrum (CD_3CN, 400 MHz, 298 K) of **3**S with assignment of signals when preparing the sample at 0.20 mM concentration of ligand $\mathbf{L}^S$.

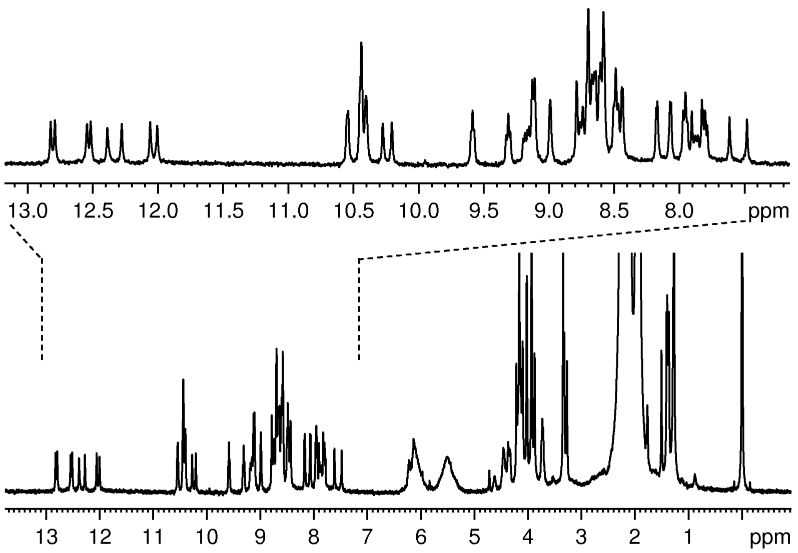

Supplementary Figure 30. ^{1}H NMR spectrum (CD_3CN, 400 MHz, 298 K) of **4**S with assignment of signals when preparing the sample at 8.40 mM concentration of ligand **L**^{S}.

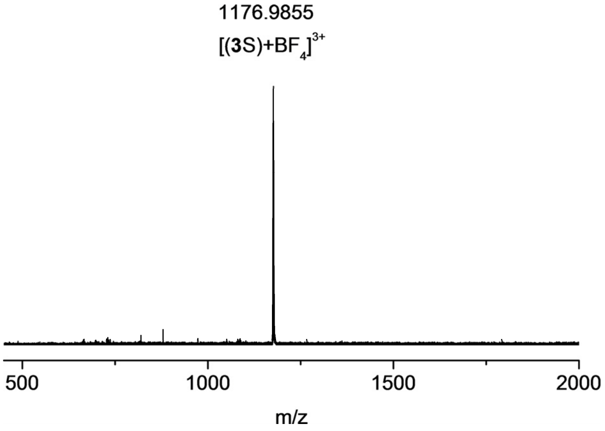

Supplementary Figure 31. Traces of the high-resolution ESI-MS of **3**S corresponding to 3+ signals. The sample was prepared with low concentration (0.20 mM for ligand concentration). The Mass spectra were recorded immediately after dilution of the sample to 0.02 mM.

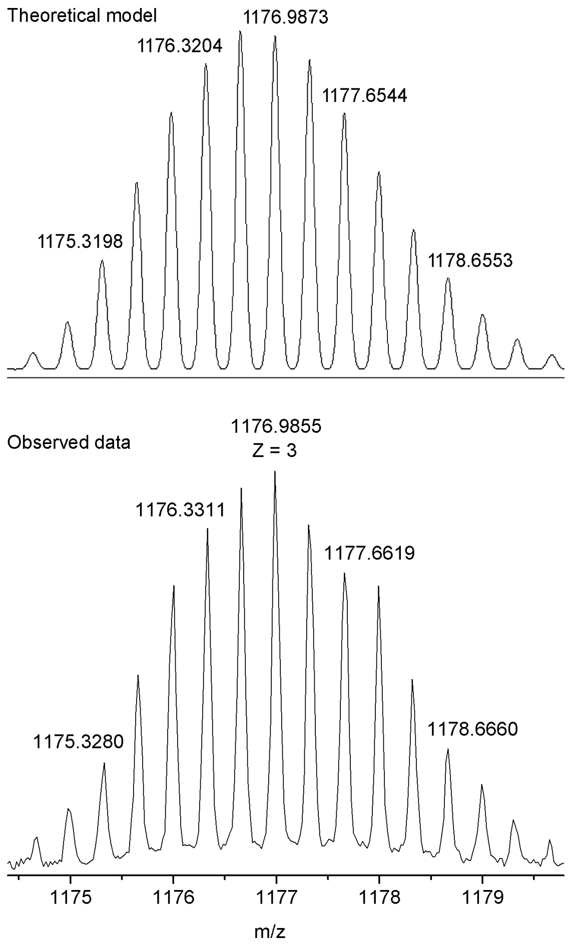

Supplementary Figure 32. High-resolution ESI-MS data for the +3 peaks of **3**S.

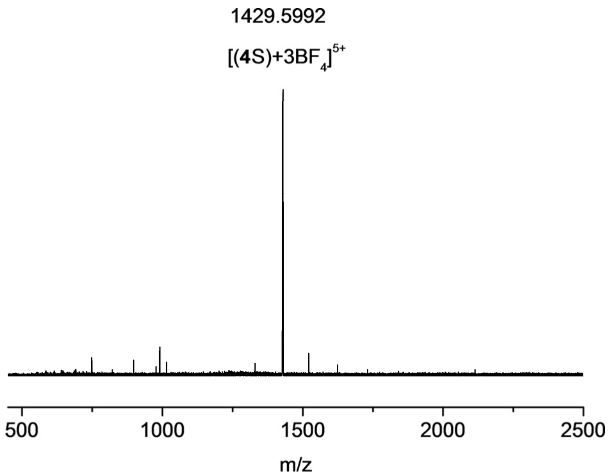

Supplementary Figure 33. Traces of the high-resolution ESI-MS of **4**S corresponding to 5+ signals. The sample was prepared with high concentration (6.00 mM for ligand concentration). The Mass spectra were recorded immediately after dilution of the sample to 0.02 mM.

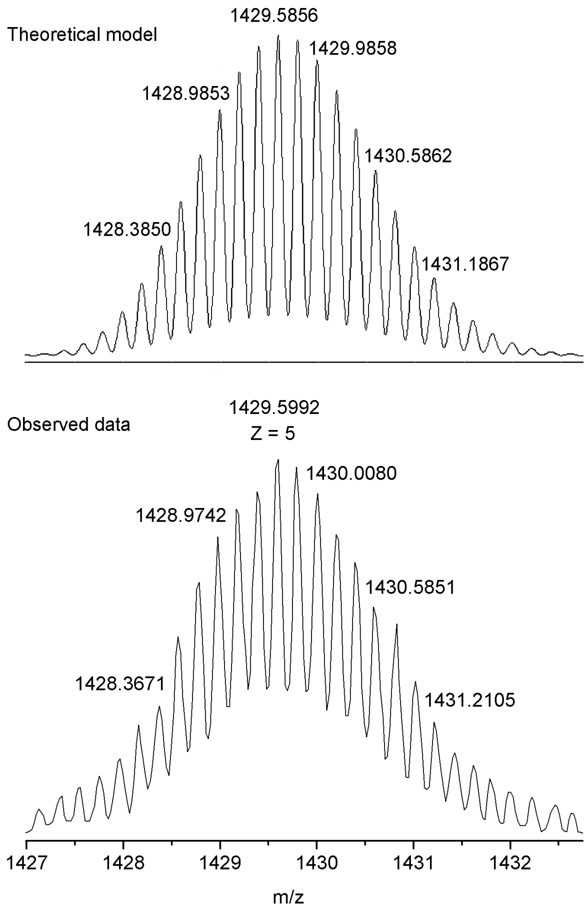

Supplementary Figure 34. High-resolution ESI-MS data for the +5 peaks of **4**S.

8.2 Self-assembly of the complexes [2Cl⊂**4**R] and [2Cl⊂**4**S]

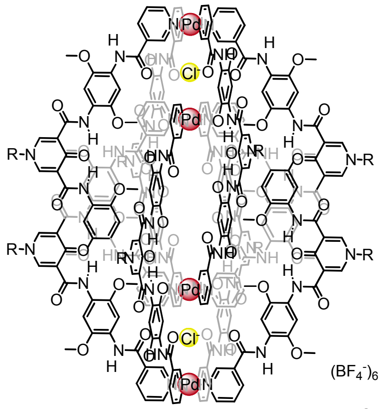

[2Cl⊂**4**R]: [2Cl+($Pd_4L^R{}_8$)], R = 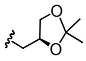

[2Cl⊂**4**S]: [2Cl+($Pd_4L^S{}_8$)], R = 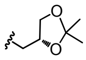

The complex [2Cl⊂**4**R] (or [2Cl⊂**4**S]) were formed by the mixture solution of monomeric helicate **3**R and dimeric helicate **4**R (or **3**S and **4**S) which obtained above with NBu_4Cl (0.70 μmol, 40 μL of a 17.5 mM stock solution in CD_3CN) at 56 °C for 12 h in a NMR tube to give a 0.32 mM solution of the complex [2Cl⊂**4**R] (or [2Cl⊂**4**S]). ^1H NMR (CD_3CN, 400 MHz): δ 12.35 (s, 8 H), 11.92 (s, 16 H), 11.38 (s, 8 H), 10.42 (s, 8 H), 9.50 (s, 8 H), 8.75 (s, 8 H), 8.67 - 8.62 (m, 16 H), 8.53 - 8.49 (m, 16 H), 8.40 (d, J = 7.36 Hz, 8 H), 8.20 (s, 8 H), 8.10 (s, 8 H), 8.06 (d, J = 7.24 Hz, 8 H), 7.83 (t, J = 5.64 Hz, 8 H), 7.53 - 7.45 (m, 8 H), 7.24 (s, 8 H), 4.61 - 4.53 (m, 8 H), 4.37 - 4.31 (m, 8 H), 4.19 - 4.08 (m, 24 H), 3.89 (s, 24 H), 3.74 (s, 24 H), 3.44 (s, 24 H), 2.54 (s, 24 H), 1.41 - 1.36 (m, 48 H). ESI-MS: *m/z* 1409.1925 [(2Cl⊂**4**S)+BF_4]$^{5+}$, 1782.9573 [(2Cl⊂**4**S)+2(BF_4)]$^{4+}$.

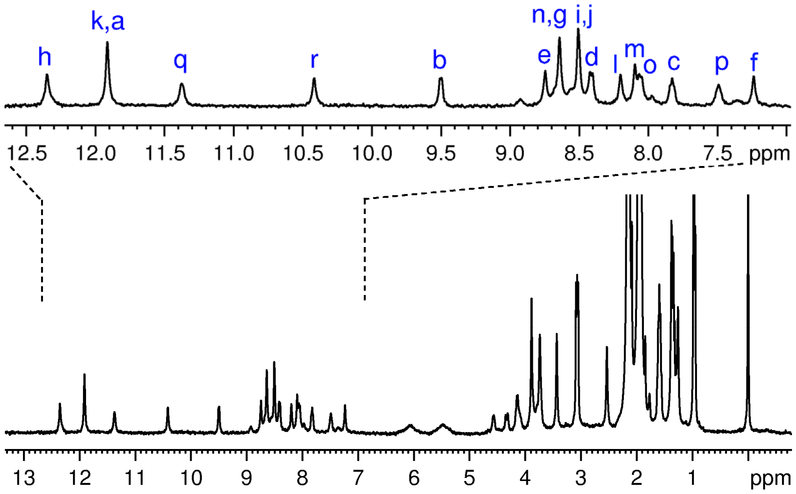

Supplementary Figure 35. ^1H NMR spectrum (CD_3CN, 400 MHz, 298 K) of [2Cl⊂**4**S] with assignment of signals.

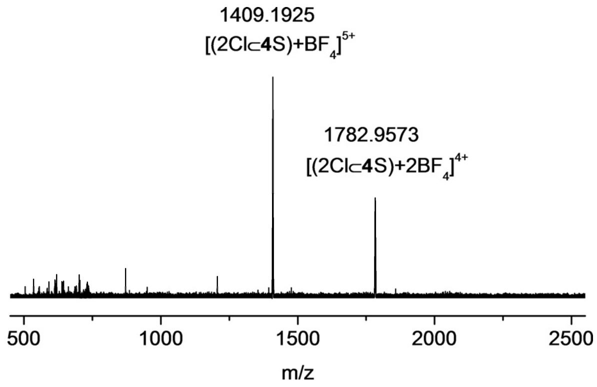

Supplementary Figure 36. Traces of the high-resolution ESI-MS of [2Cl⊂**4**S] corresponding to 4+ and 5+ signals.

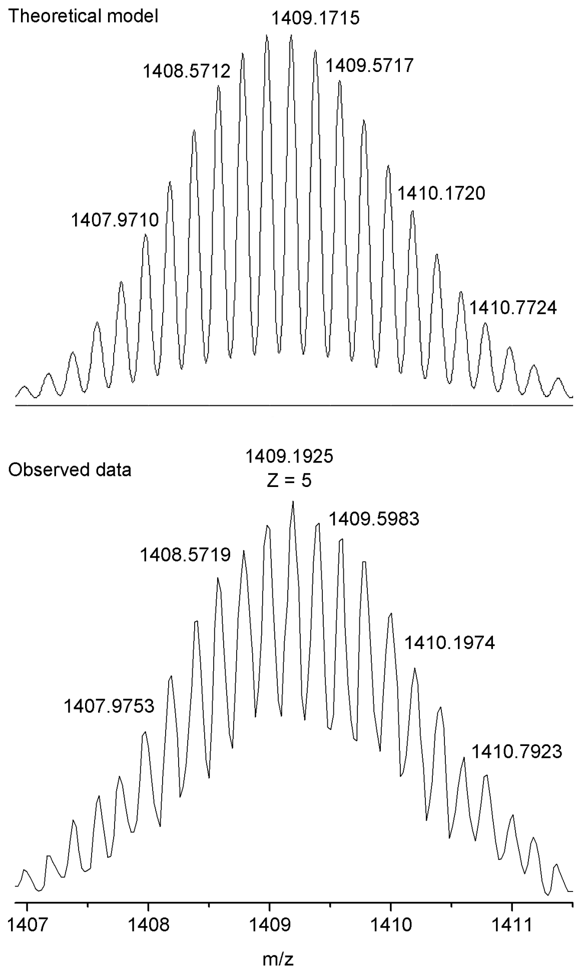

Supplementary Figure 37. High-resolution ESI-MS data for the +5 peaks of [2Cl⊂**4**S].

8.3 Amplification of chirality of the dimeric helicates

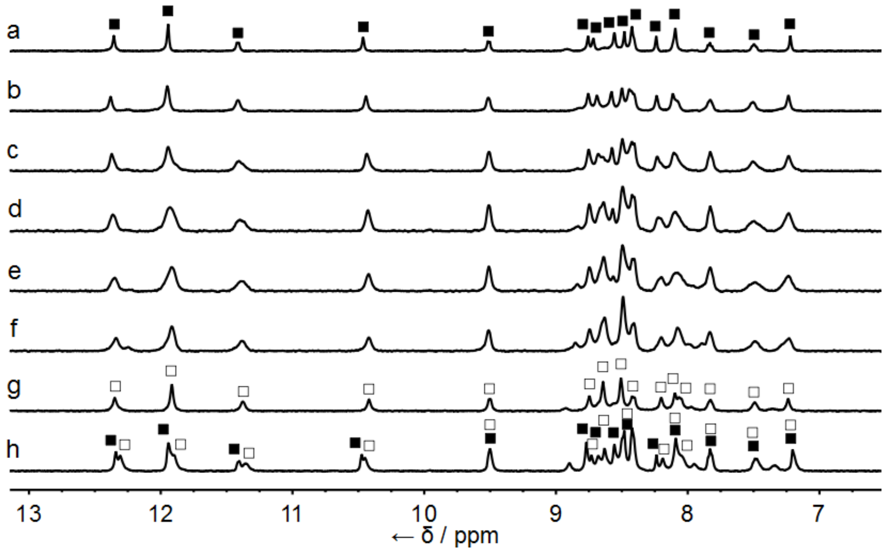

Supplementary Figure 38. Representative ^{1}H NMR spectrum (CD_3CN, 400 MHz, 298 K) of mixtures of [2Cl⊂**2**] and [2Cl⊂**4**S] (ca. 2[2Cl⊂**2**] + 2[2Cl⊂**4**S] = 0.60 mM) in different proportions equilibrated at 56 °C: (a) 10 : 0, (b) 9 : 1, (c) 7 : 3, (d) 5 : 5, (e) 3 : 7, (f) 1 : 9, (g) 0 : 10, and (h) recorded immediately after mixing [2Cl⊂**2**] and [2Cl⊂**4**S] (1 : 1). Signals of [2Cl⊂**2**] and of [2Cl⊂**4**S] are marked with black and empty white squares, respectively. And CD spectra of these complexes are showed in Figure 5b. The broadening of signals indicates a purely statistical mixture of the heteromeric complex through the ligand exchange process.

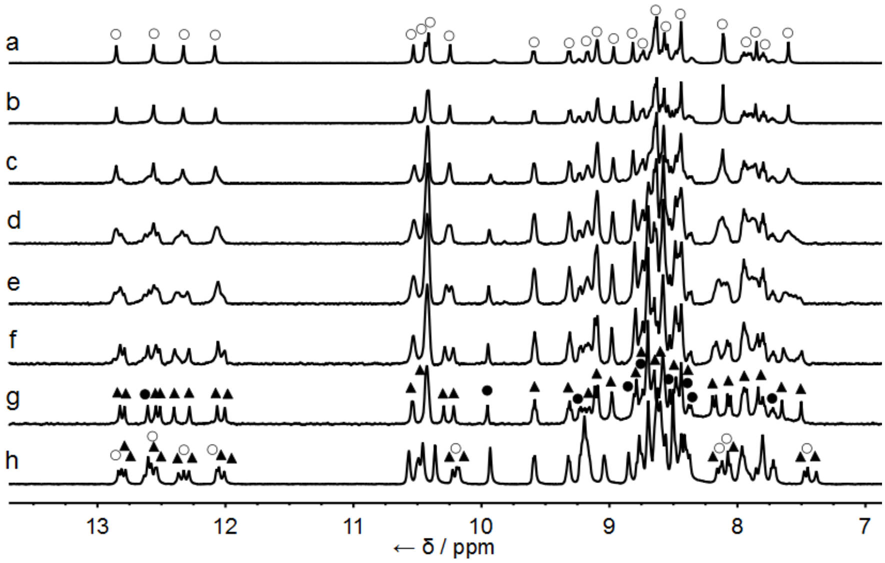

Supplementary Figure 39. Representative ^{1}H NMR spectrum (CD_3CN, 400 MHz, 298 K) of the mixture of **2** and **4**S (ca. [**1**] + 2[**2**] + [**3**S] + 2[**4**S] = 1.20 mM) in different proportions equilibrated at 56 °C: (a) 10 : 0, (b) 9 : 1, (c) 7 : 3, (d) 5 : 5, (e) 3 : 7, (f) 1 : 9, (g) 0 : 10, and (h) recorded immediately after mixing **2** and **4**S (1 : 1). Signals of the monomeric helicate are marked with black circles. Signals of **2** and of **4**S are marked with empty white circles and black triangles, respectively. And CD spectra of these complexes are showed in Figure 5c.

9 Supplementary NMR spectra

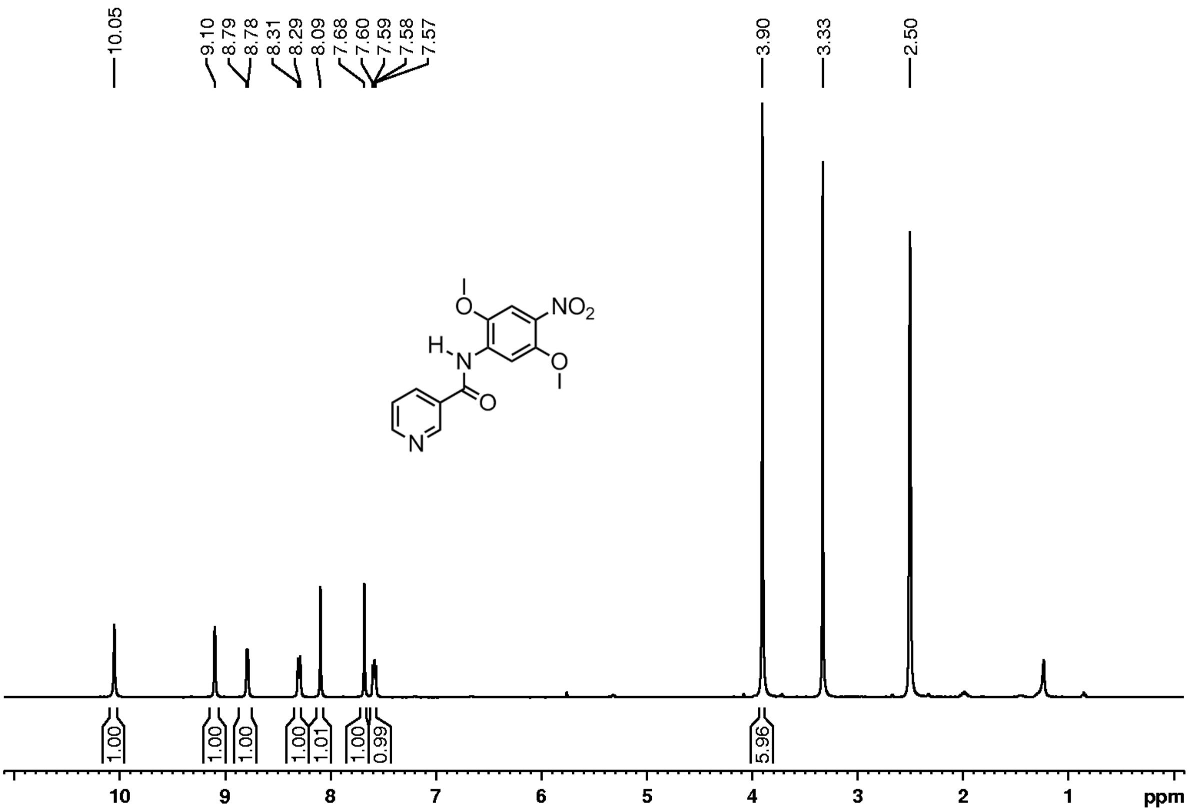

Supplementary Figure 40. ^{1}H NMR spectrum (400 MHz) of compound **6** in DMSO-d$_6$.

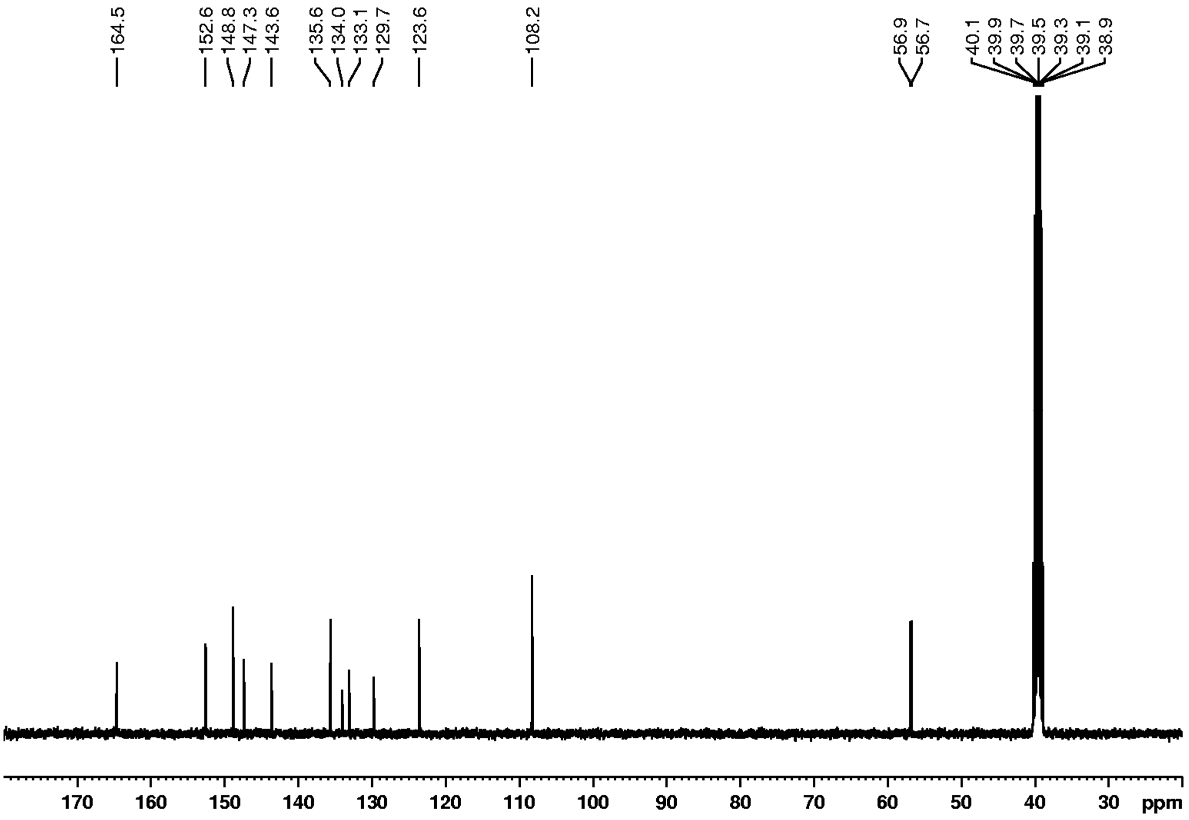

Supplementary Figure 41. ^{13}C NMR spectrum (100 MHz) of compound **6** in DMSO-d$_6$.

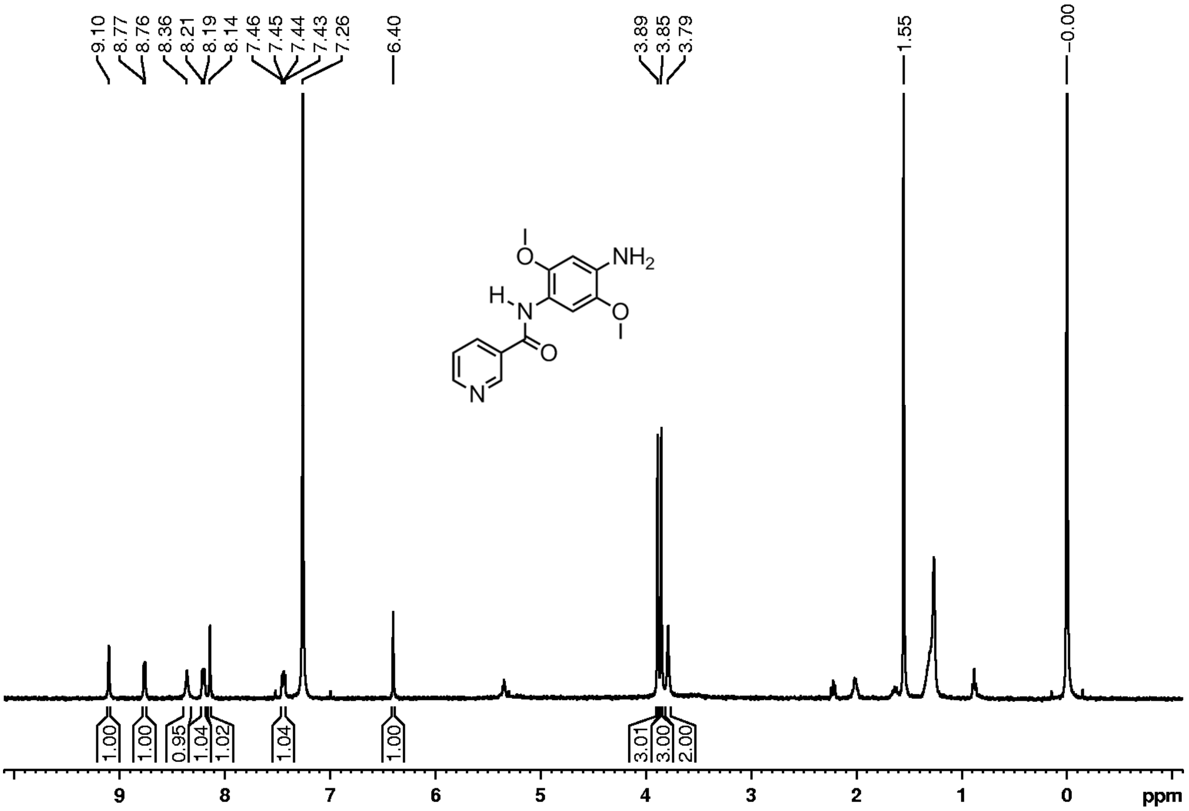

Supplementary Figure 42. ^{1}H NMR spectrum (400 MHz) of compound **7** in $CDCl_3$.

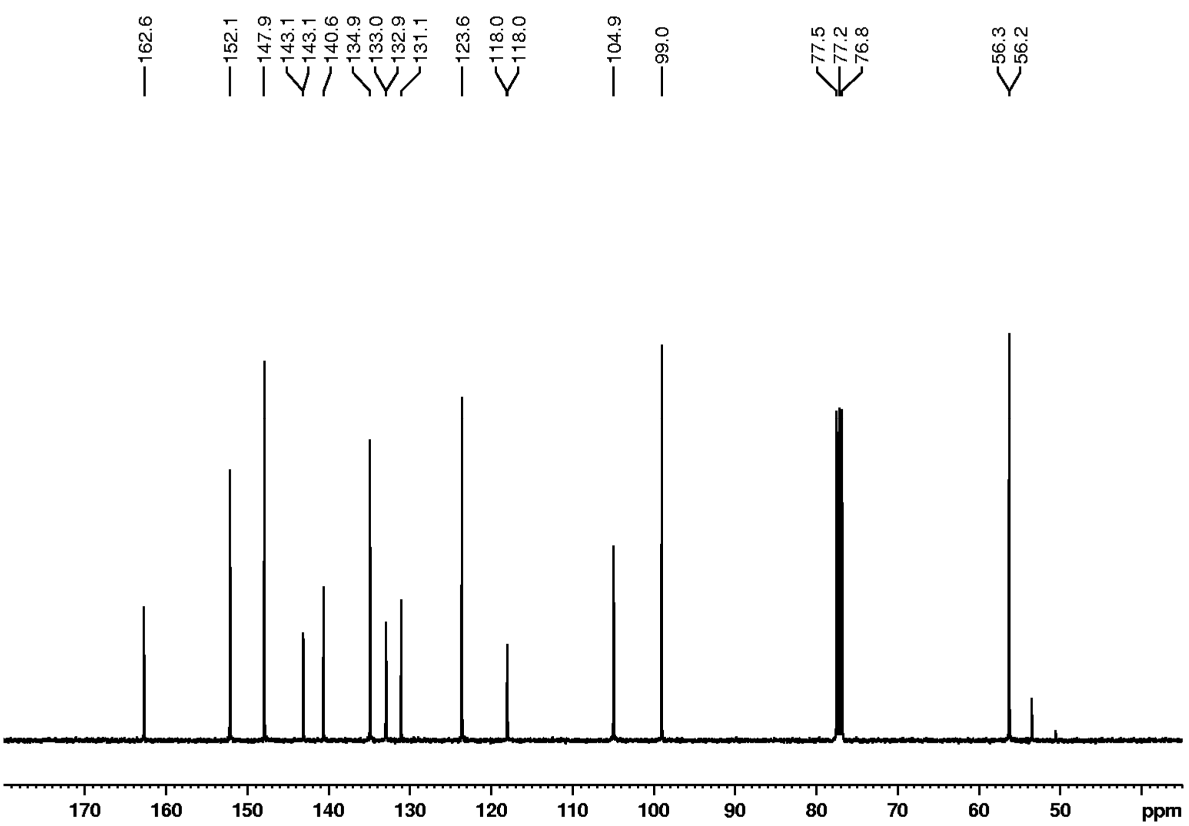

Supplementary Figure 43. ^{13}C NMR spectrum (100 MHz) of compound **7** in $CDCl_3$.

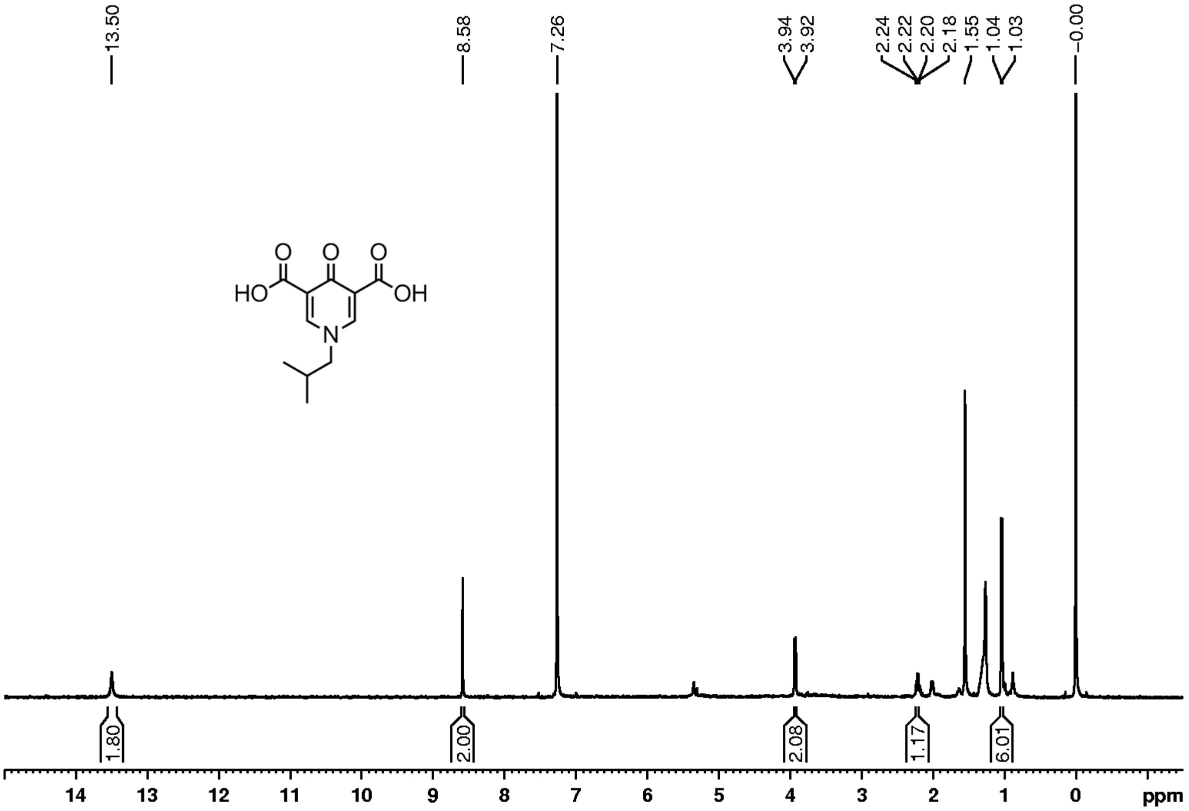

Supplementary Figure 44. ^{1}H NMR spectrum (400 MHz) of compound **9** in $CDCl_3$.

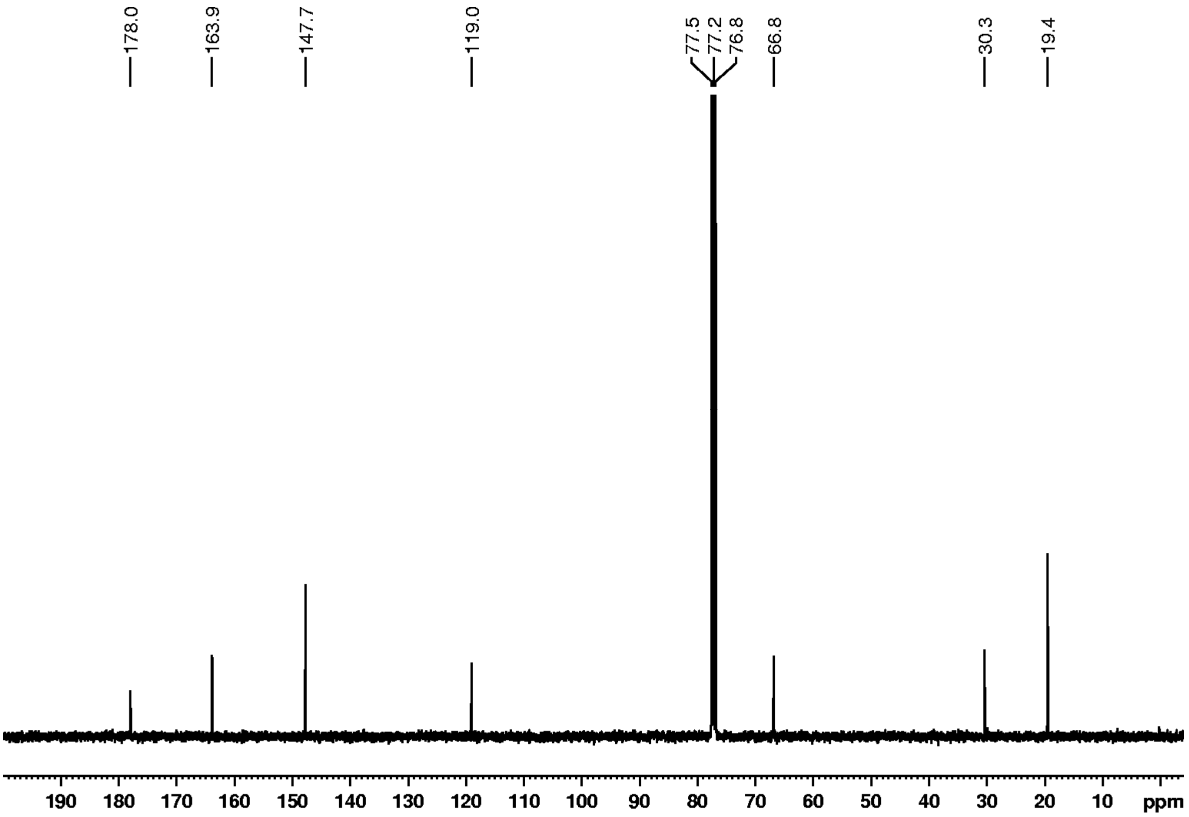

Supplementary Figure 45. ^{13}C NMR spectrum (100 MHz) of compound **9** in $CDCl_3$.

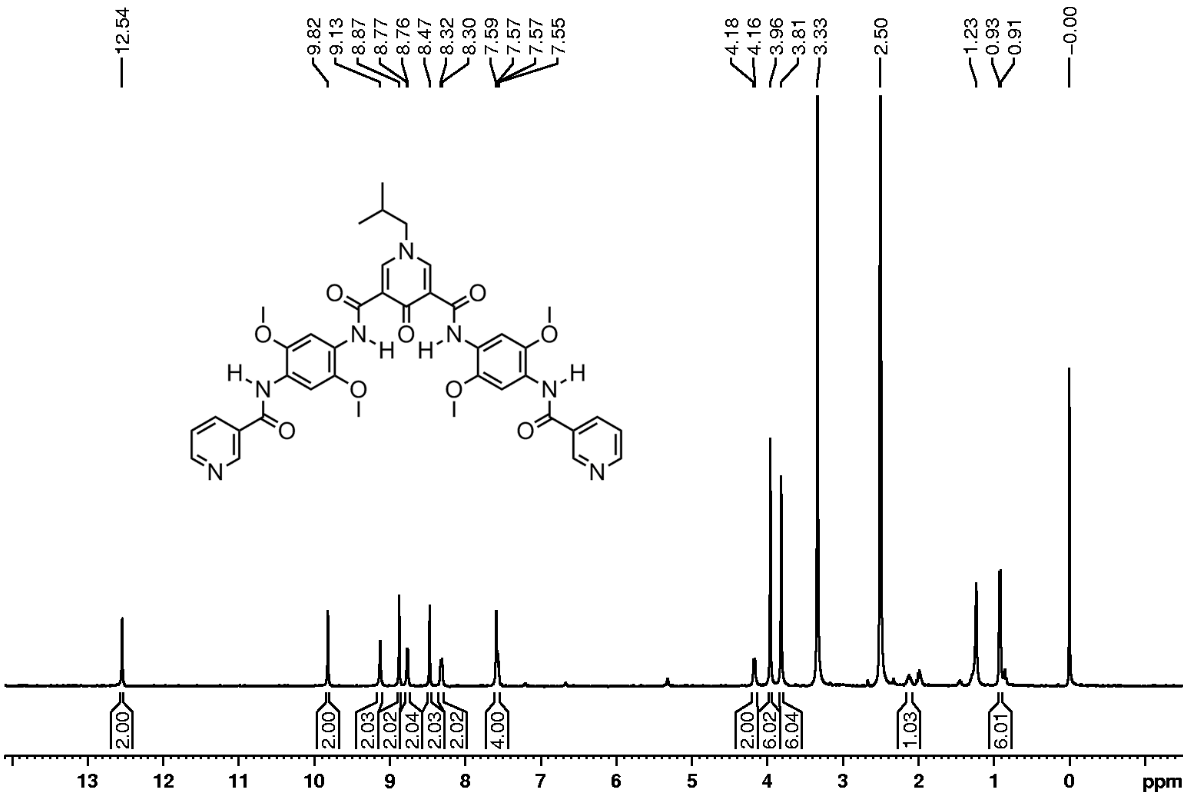

Supplementary Figure 46. ^{1}H NMR spectrum (400 MHz) of ligand **L** in DMSO-d_6.

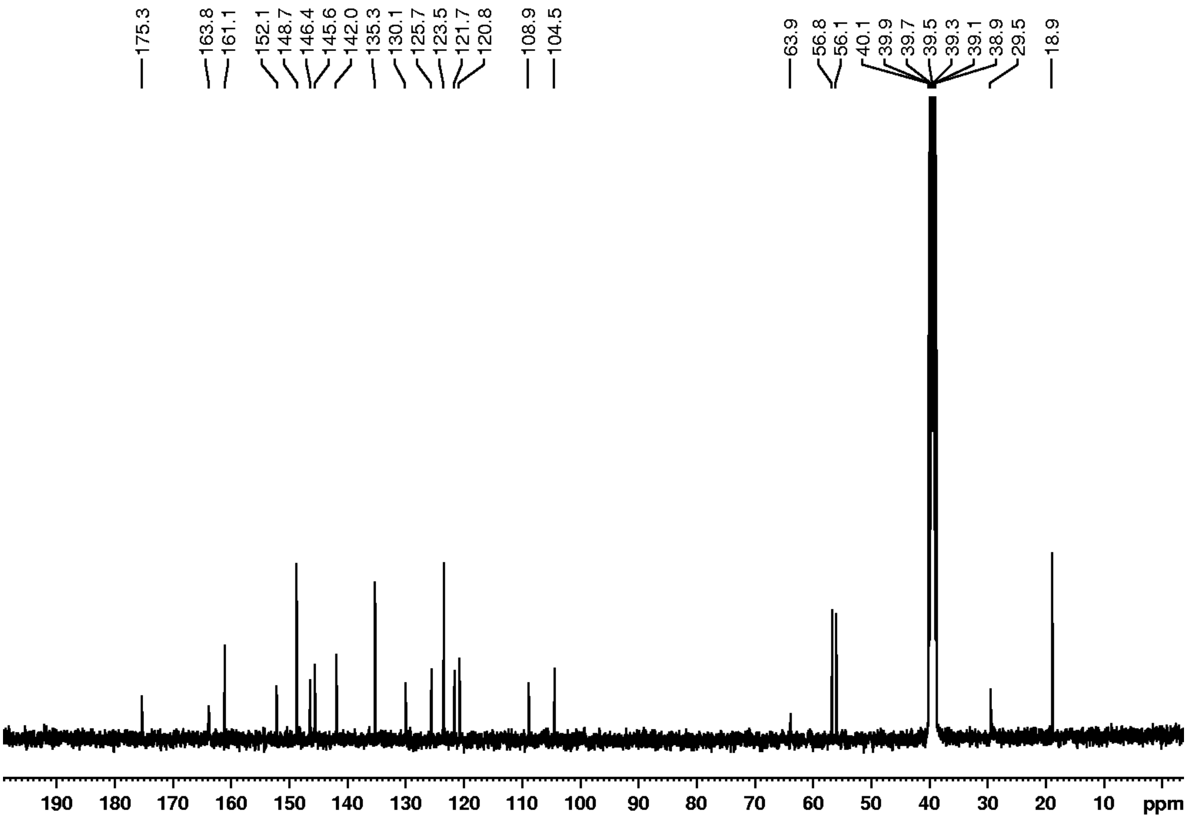

Supplementary Figure 47. ^{13}C NMR spectrum (100 MHz) of ligand **L** in DMSO-d_6.

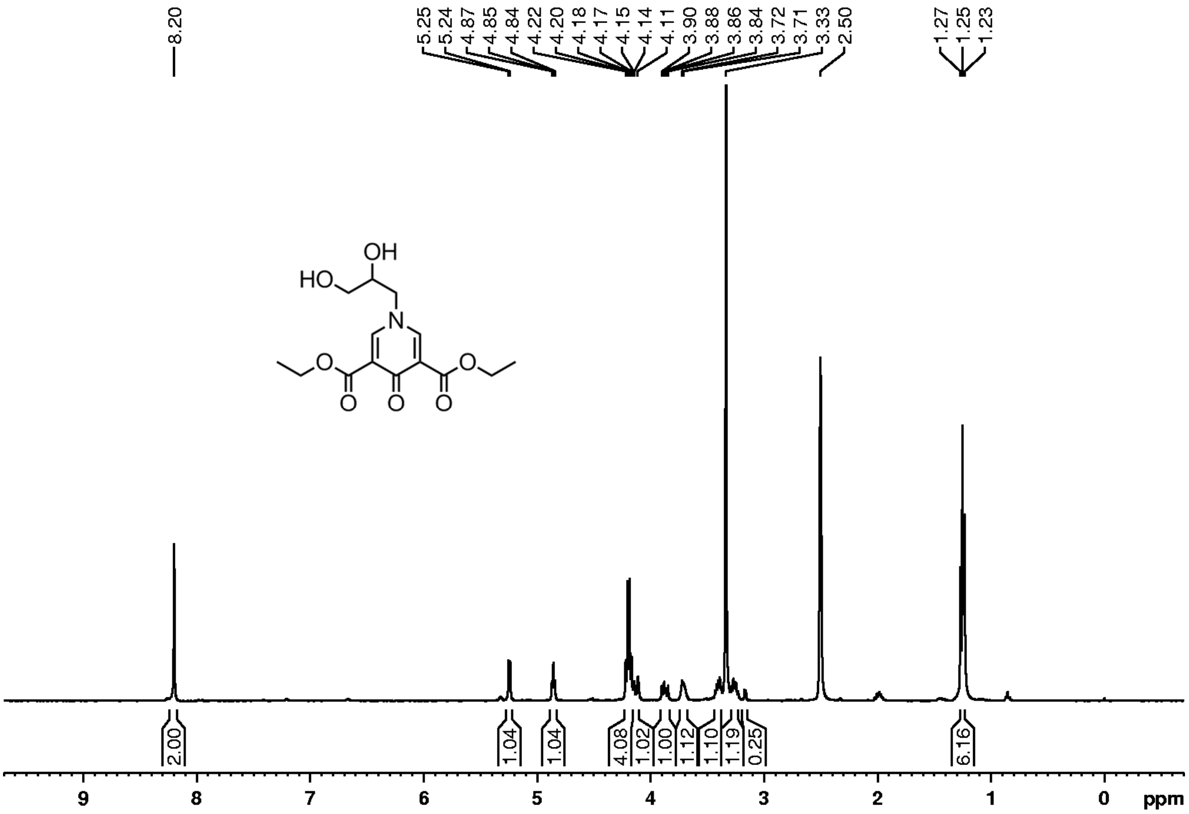

Supplementary Figure 48. ^{1}H NMR spectrum (400 MHz) of compound **11** in DMSO-d_6.

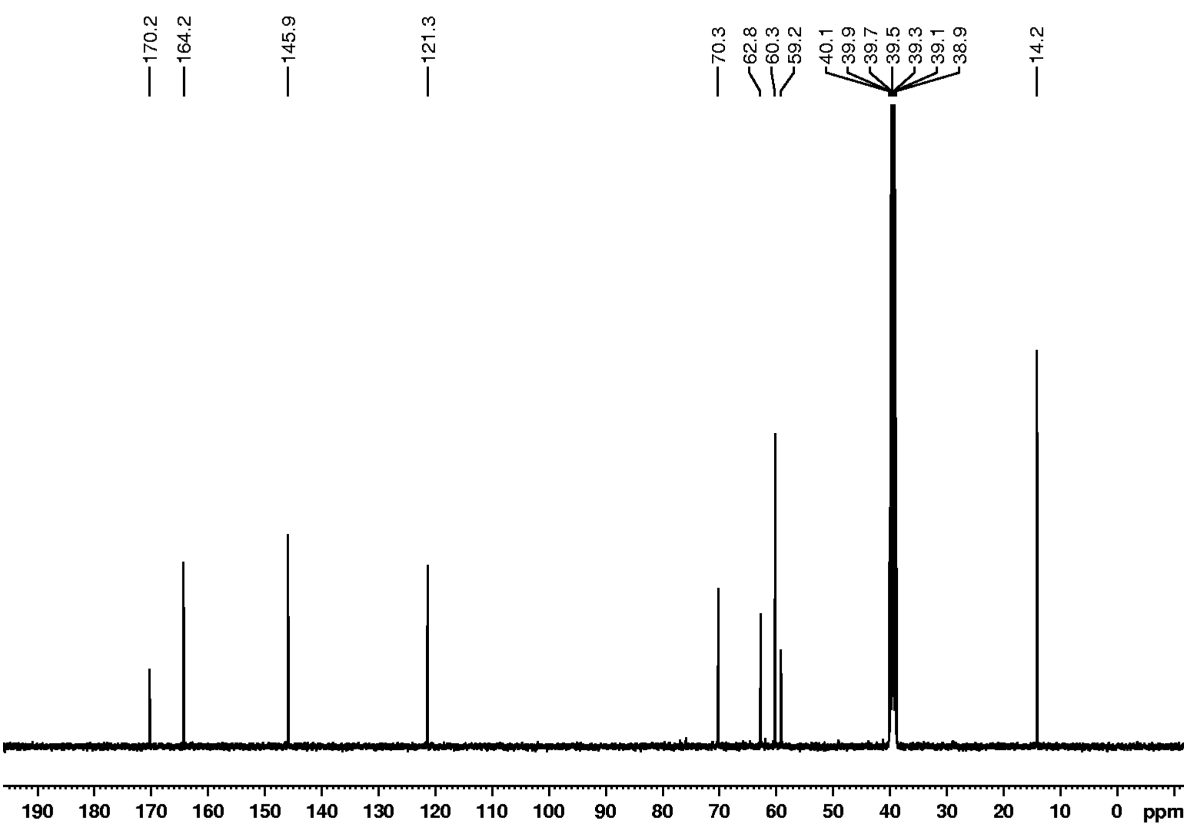

Supplementary Figure 49. ^{13}C NMR spectrum (100 MHz) of compound **11** in DMSO-d_6.

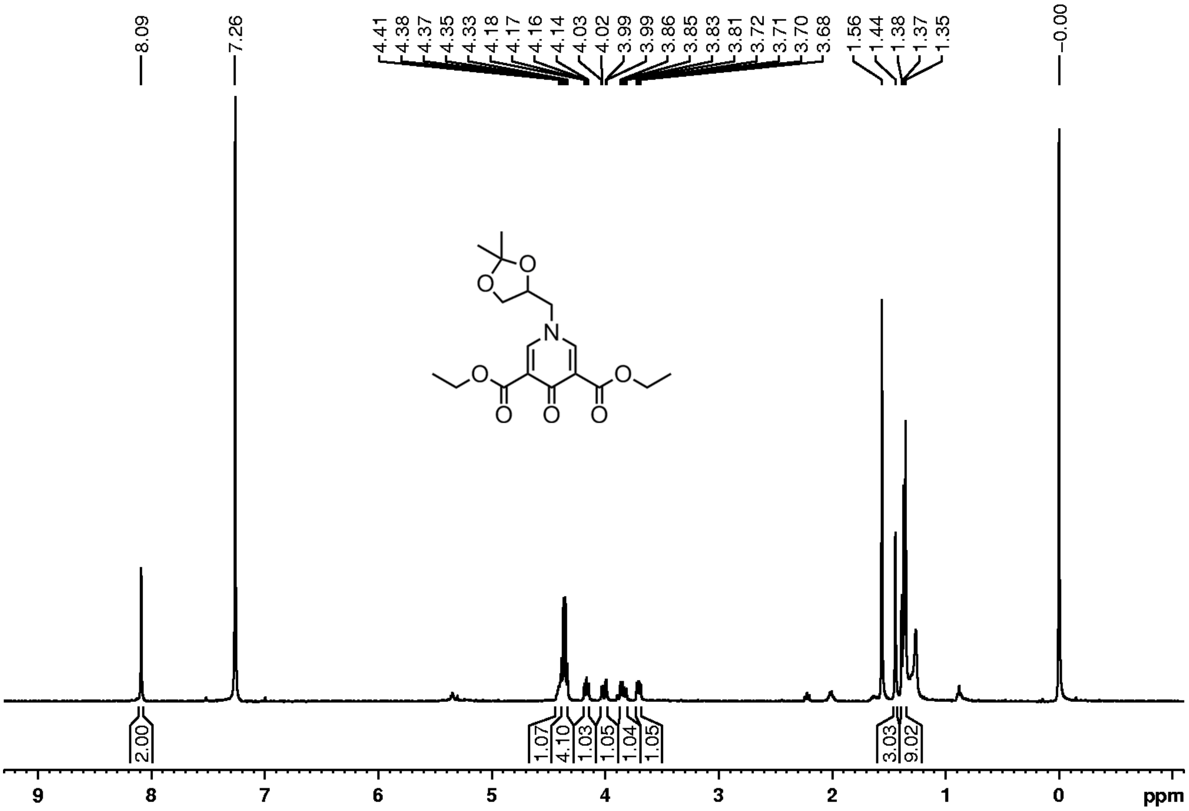

Supplementary Figure 50. ^{1}H NMR spectrum (400 MHz) of compound **12** in $CDCl_3$.

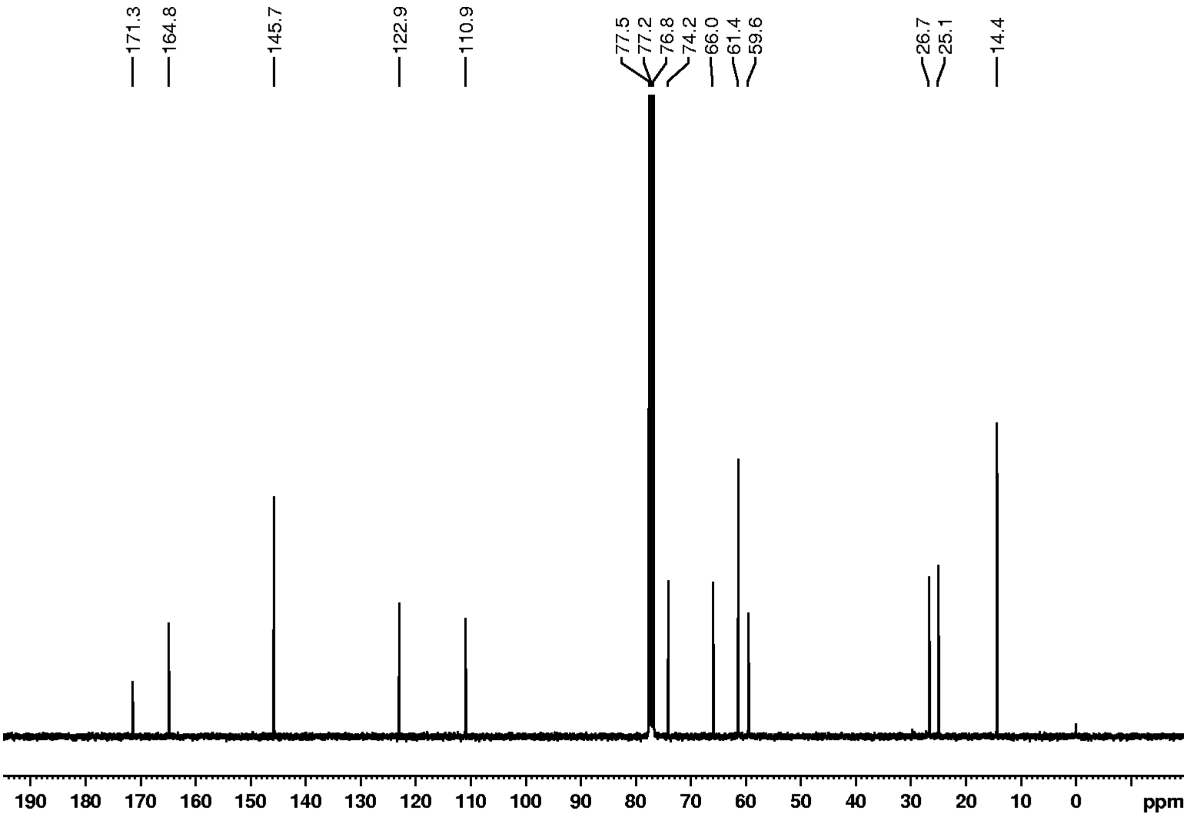

Supplementary Figure 51. ^{13}C NMR spectrum (100 MHz) of compound **12** in $CDCl_3$.

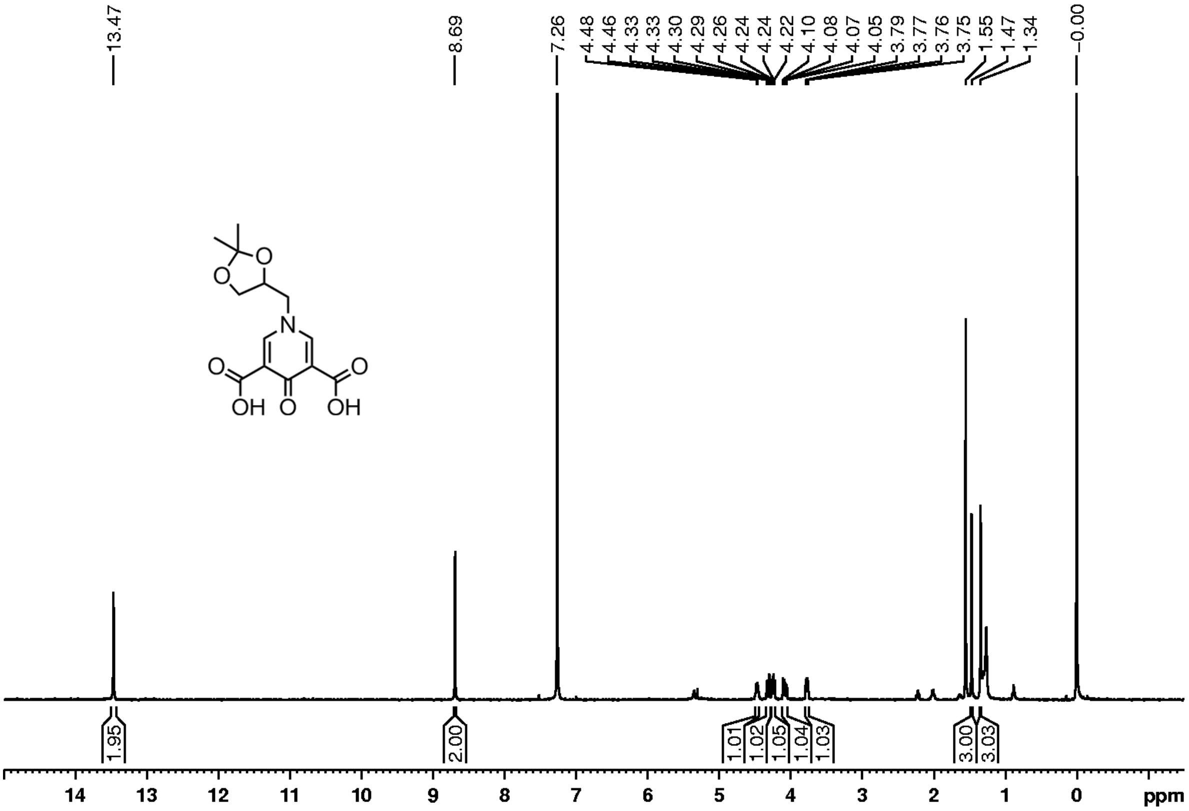

Supplementary Figure 52. ^{1}H NMR spectrum (400 MHz) of compound **13** in $CDCl_3$.

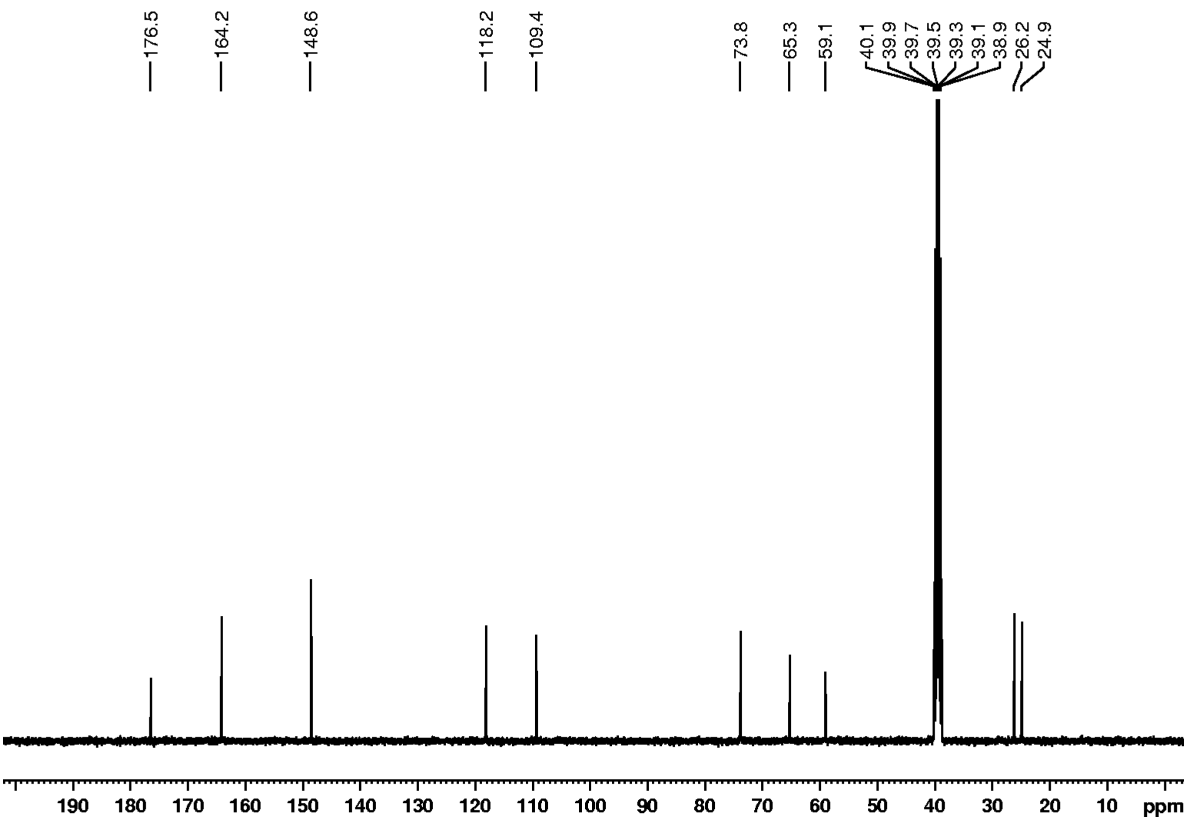

Supplementary Figure 53. ^{13}C NMR spectrum (100 MHz) of compound **13** in DMSO-d_6.

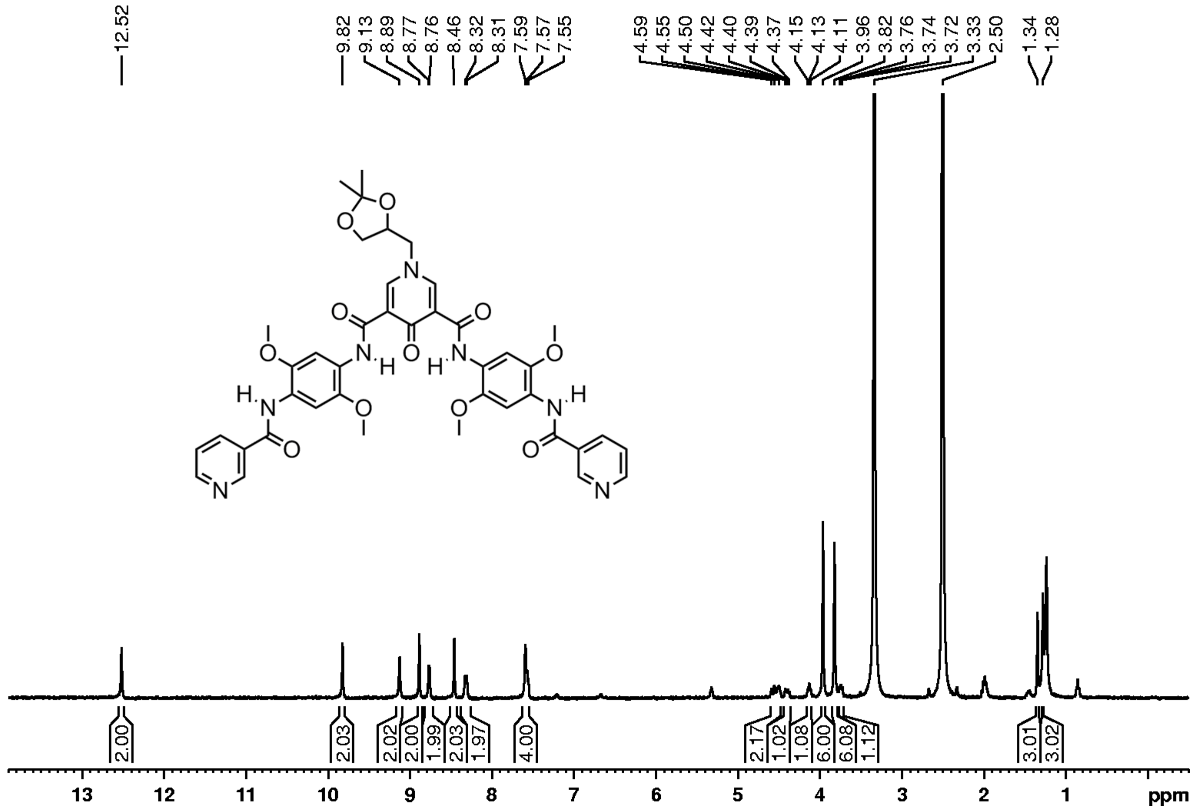

Supplementary Figure 54. ^{1}H NMR spectrum (400 MHz) of ligand $\mathbf{L}^{S}$ in DMSO-d_6.

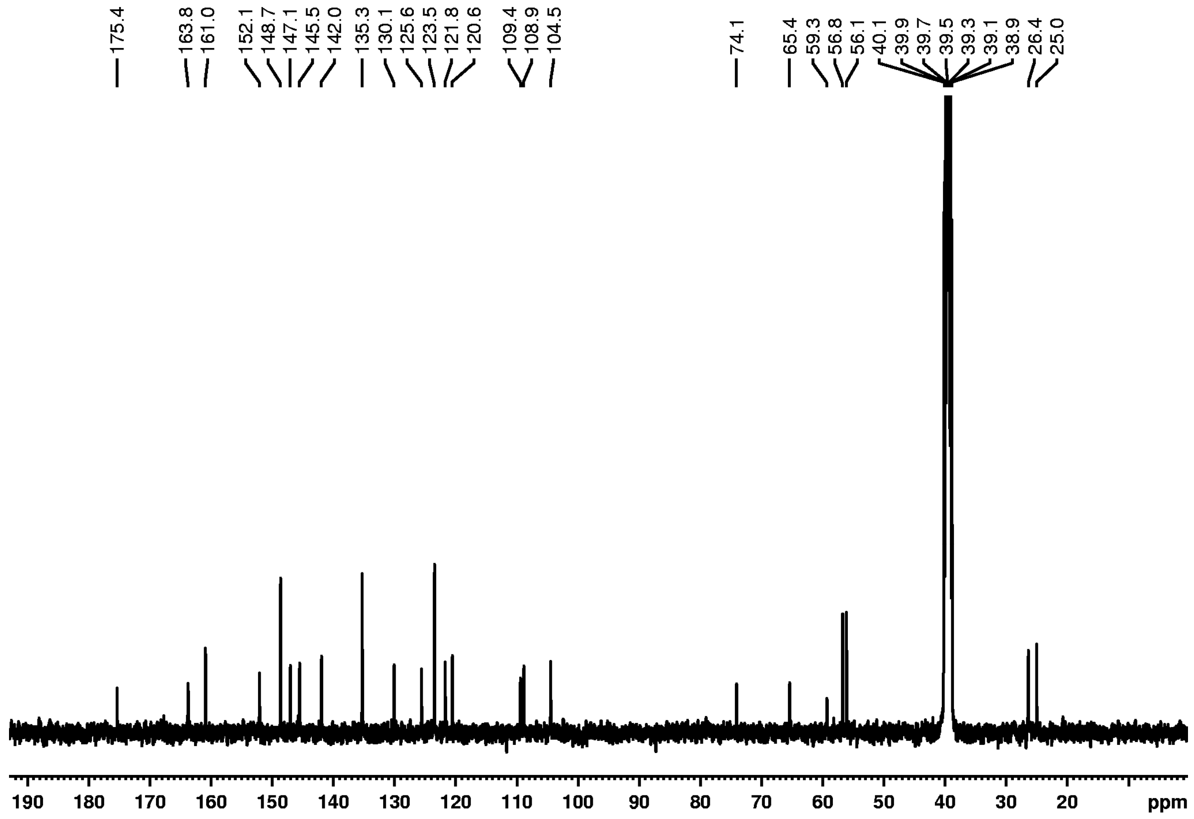

Supplementary Figure 55. ^{1}H NMR spectrum (400 MHz) of ligand $\mathbf{L}^{S}$ in DMSO-d_6.

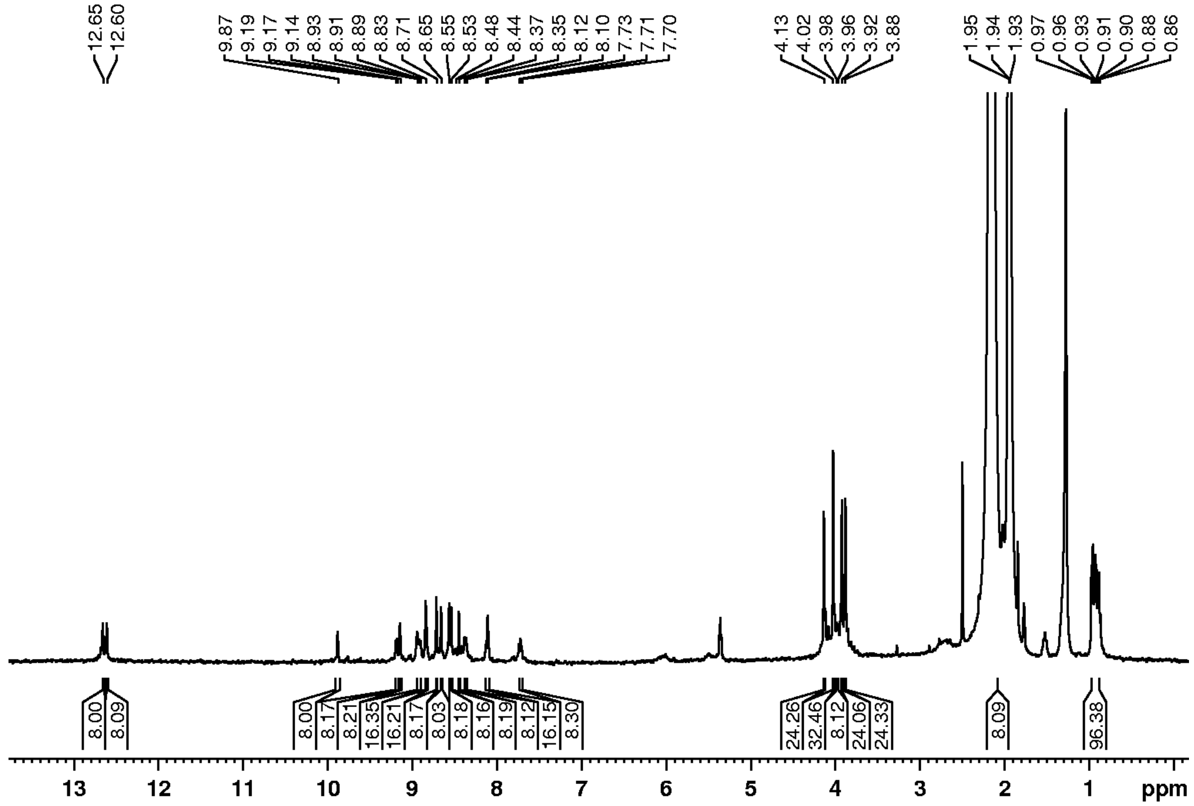

Supplementary Figure 56. ^{1}H NMR spectrum (400 MHz) of monomeric helicate **1-Pt** in CD_3CN.

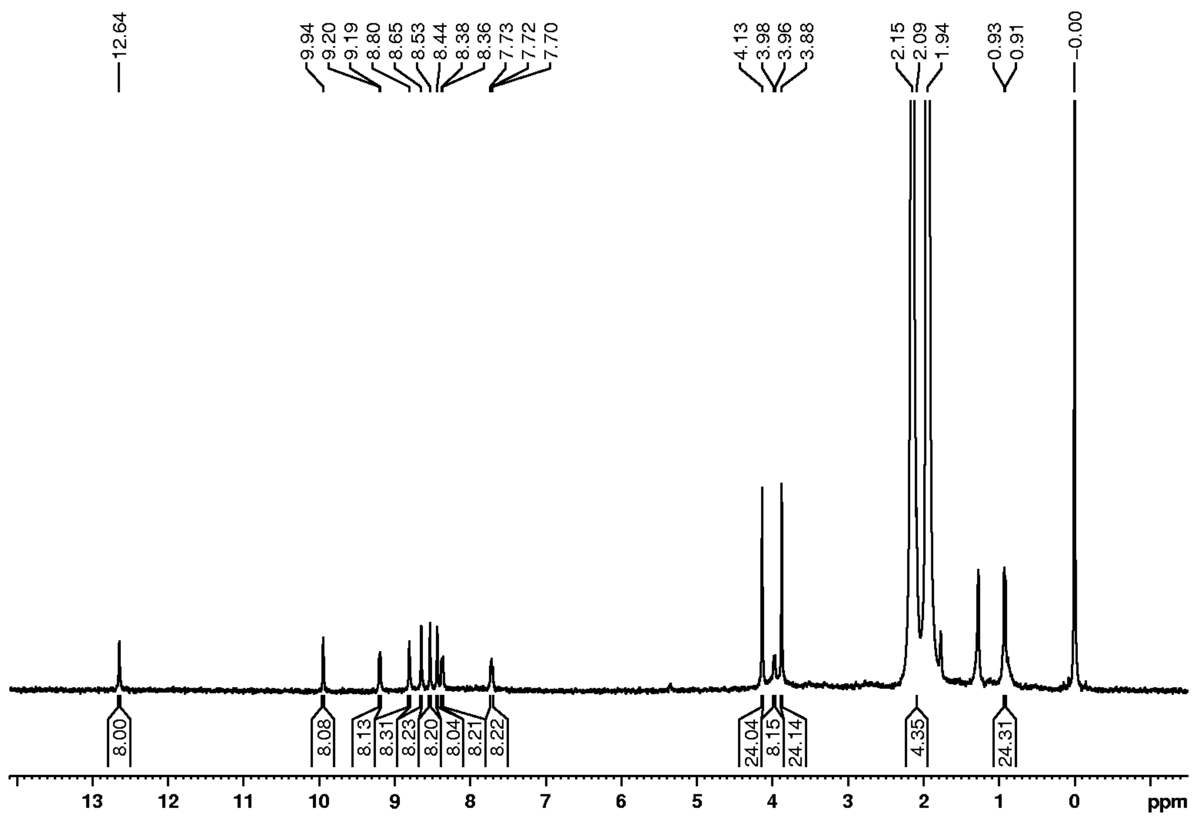

Supplementary Figure 57. ^{1}H NMR spectrum (400 MHz) of monomeric helicate **1** in CD_3CN.

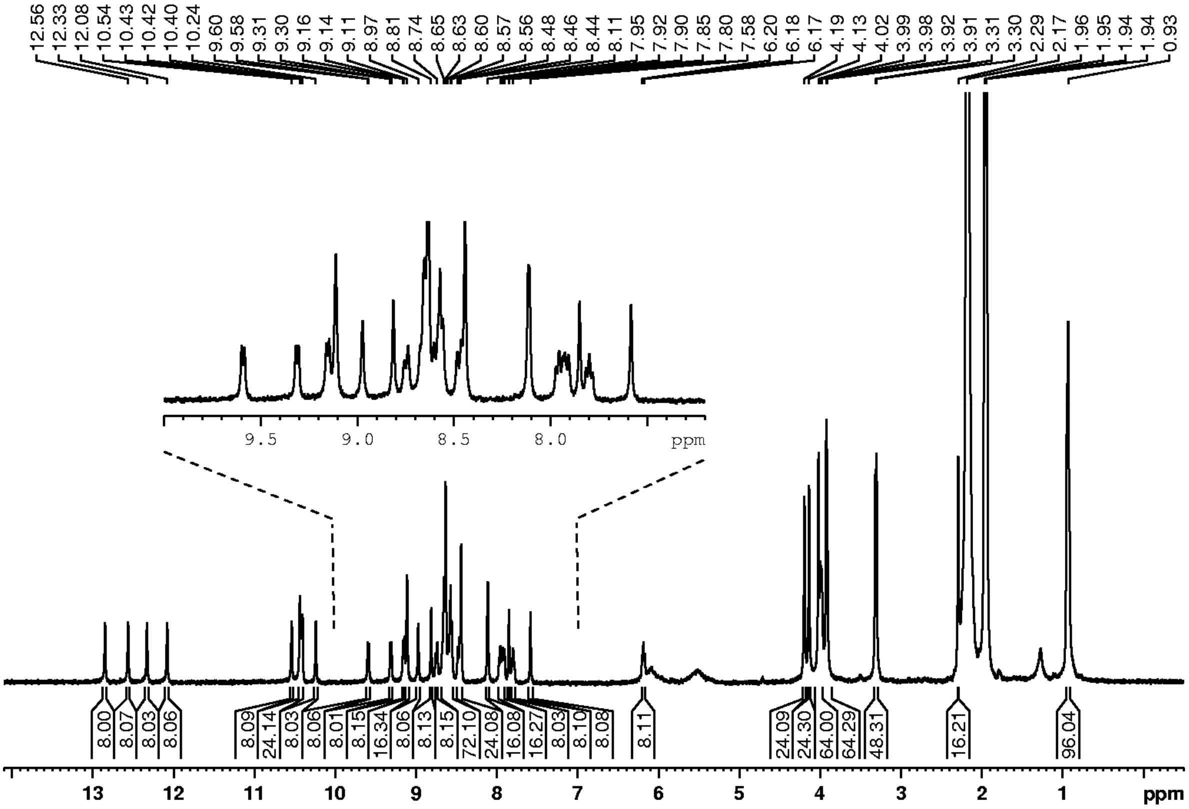

Supplementary Figure 58. ^{1}H NMR spectrum (400 MHz) of dimeric helicate **2** in CD_3CN.

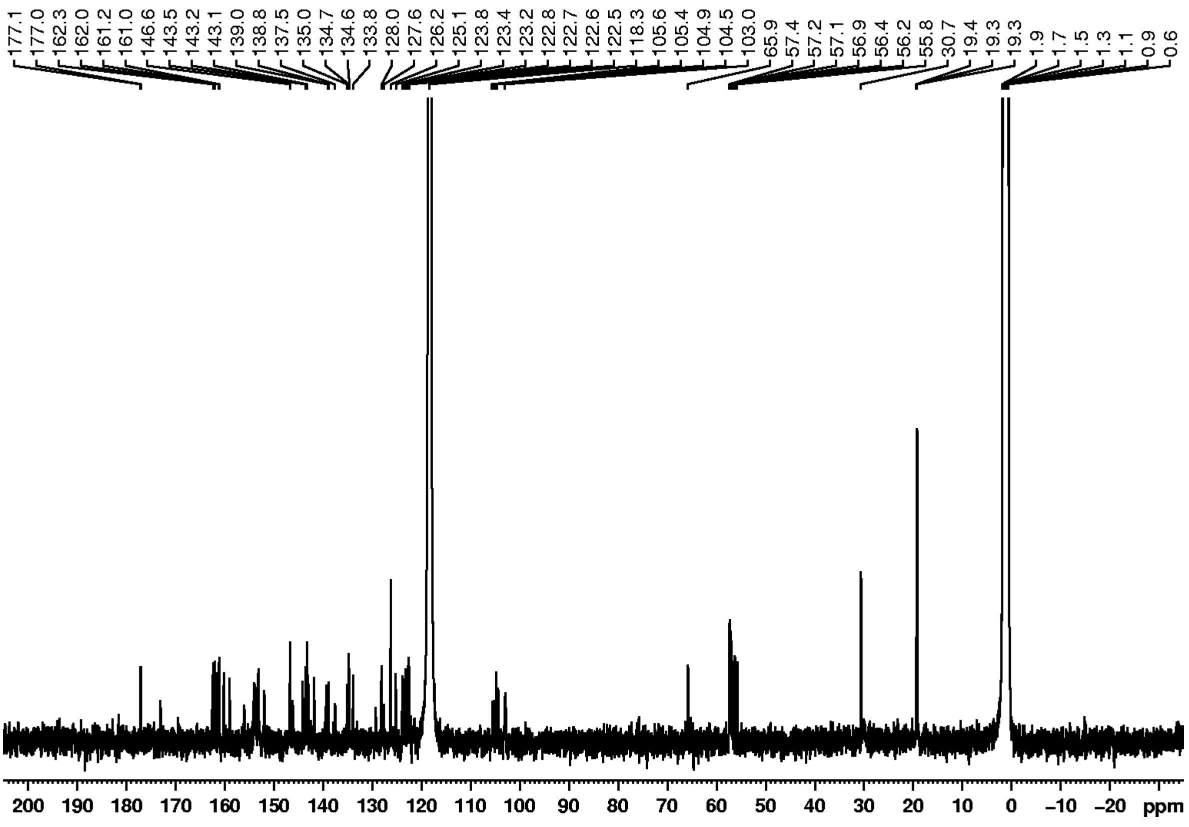

Supplementary Figure 59. ^{13}C NMR spectrum (400 MHz) of dimeric helicate **2** in CD_3CN.

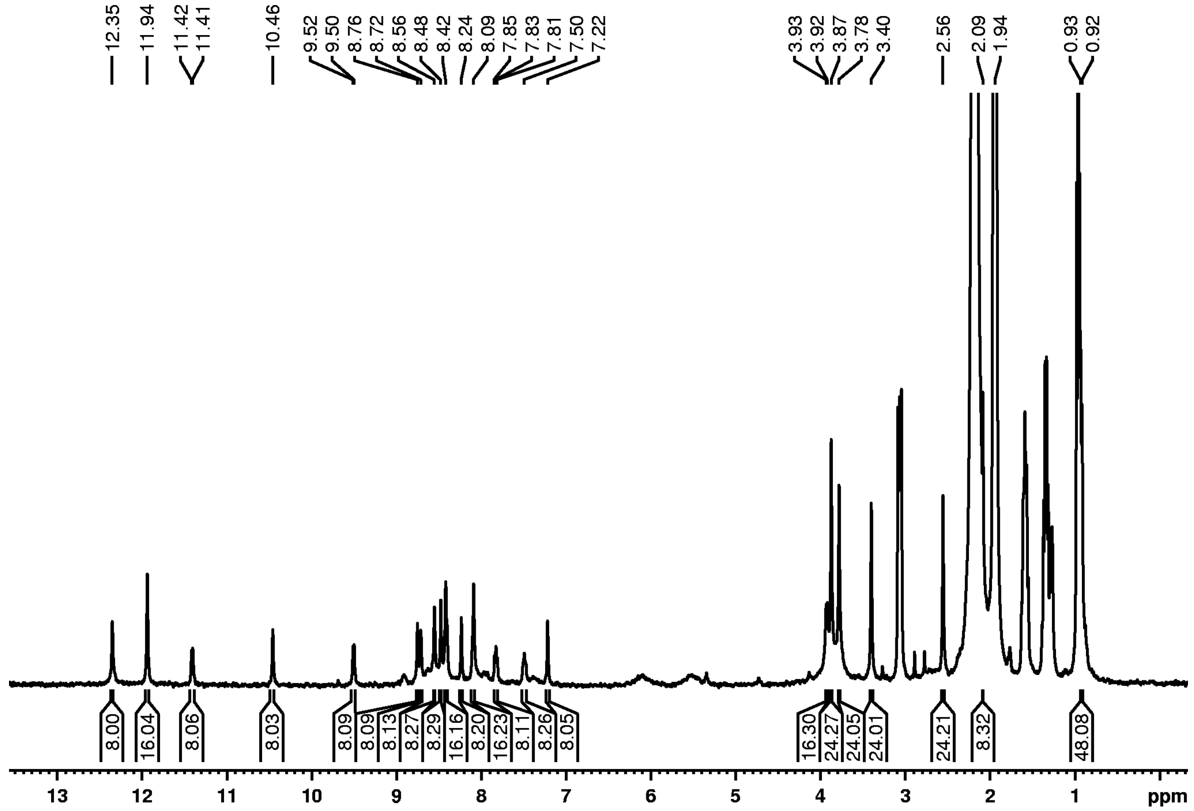

Supplementary Figure 60. ^{1}H NMR spectrum (400 MHz) of complex [2Cl⊂**2**] in CD_3CN.

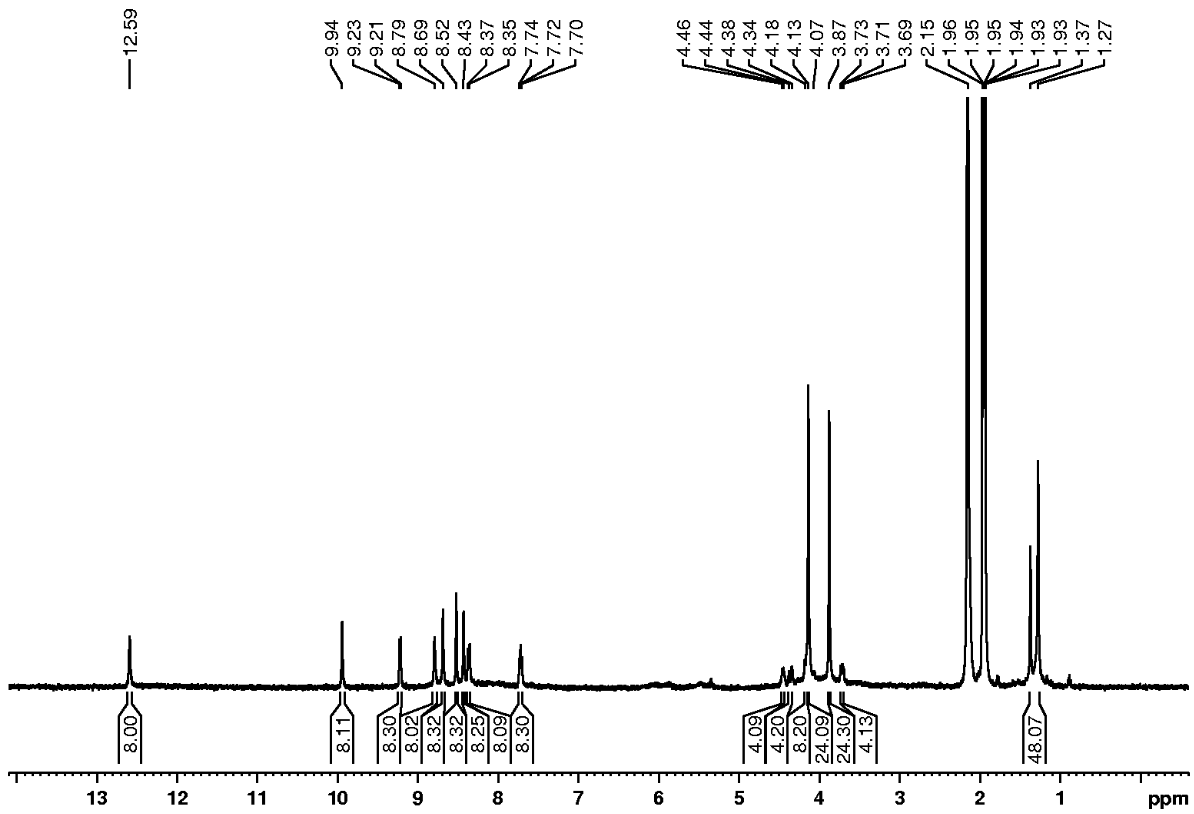

Supplementary Figure 61. ^{1}H NMR spectrum (400 MHz) of monomeric helicate **3**S in CD_3CN.

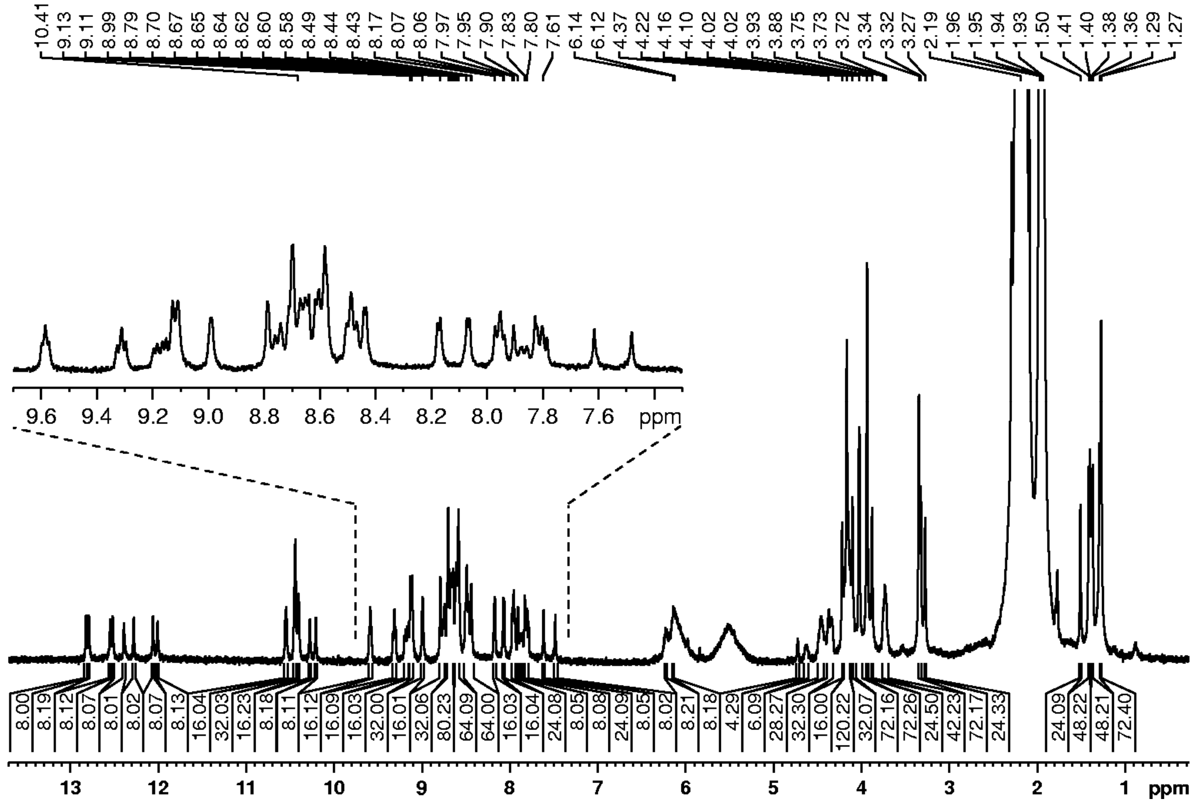

Supplementary Figure 62. ^{1}H NMR spectrum (400 MHz) of dimeric helicate **4**S in CD_3CN.

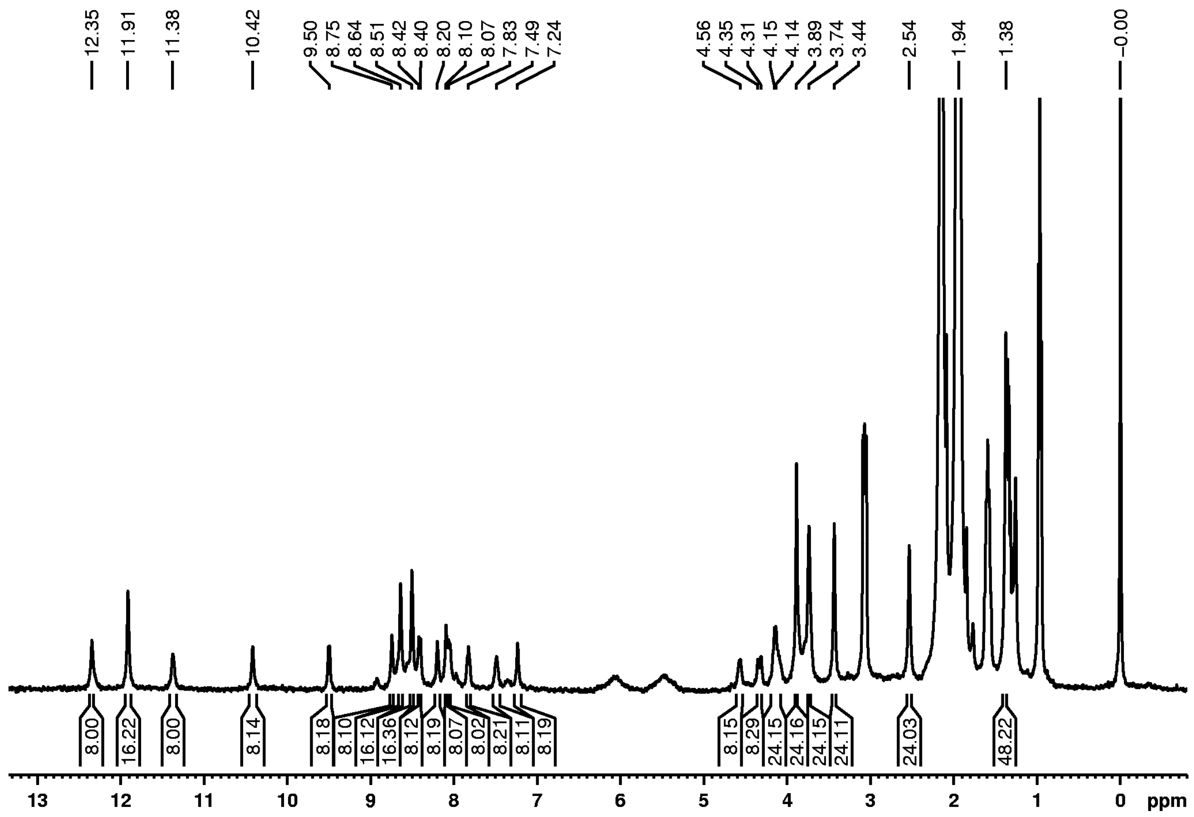

Supplementary Figure 63. ^{1}H NMR spectrum (400 MHz) of complex [2Cl⊂**4**S] in CD_3CN.

10 Supplementary References

1. Fulmer, G. R. *et al. Organometallics* **29**, 2176–2179 (2010).
2. Choi, Y., Kim, T., Jang, S., Kang, J. *New J. Chem.* **40**, 794–802 (2016).
3. Du, Z.-Y. *et al. Chem. Commun.* **47**, 12488–12490 (2011).
4. de Renzi, A. *et al. Chem. Commun.* 47–47 (1976).
5. Tripathy, D., Pal, A. K., Hanan, G. S., Chand, D. K. *Dalton Trans.* **41**, 11273–11275 (2012).
6. Spartan'14, Wavefunction Inc., Irvine CA, **2014**.
7. Frisch, M. J., Trucks, G. W., Schlegel, H. B., Scuseria, G. E., Robb, M. A., Cheeseman, J. R., Scalmani, G., Barone, V., Mennucci, B., Petersson, G. A., Nakatsuji, H., Caricato, M., Li, X., Hratchian, H. P., Izmaylov, A. F., Bloino, J., Zheng, G., Sonnenberg, J. L., Hada, M., Ehara, M., Toyota, K., Fukuda, R., Hasegawa, J., Ishida, M., Nakajima, T., Honda, Y., Kitao, O., Nakai, H., Vreven, T., J. A., Montgomery, Jr., Peralta, J. E., Ogliaro, F., Bearpark, M., Heyd, J. J., Brothers, E., Kudin, K. N., Staroverov, V. N., Kobayashi, R., Normand, J., Raghavachari, K., Rendell, A., Burant, J. C., Iyengar, S. S., Tomasi, J., Cossi, M., Rega, N., Millam, J. M., Klene, M., Knox, J. E., Cross, J. B., Bakken, V., Adamo, C., Jaramillo, J., Gomperts, R., Stratmann, R. E., Yazyev, O., Austin, A. J., Cammi, R., Pomelli, C., Ochterski, J. W., Martin, R. L., Morokuma, K., Zakrzewski, V. G., Voth, G. A., Salvador, P., Dannenberg, J. J., Dapprich, S., Daniels, A. D., Farkas, O., Foresman, J. B., Ortiz, J. V., Cioslowski, J., Fox, D. J. Gaussian 09w (version 7.0), Gaussian, Inc., Wallingford, CT, **2009**.
8. Sheldrick, G. M. SHELXT-Integrated space-group and crystal-structure determination. *Acta Cryst.* **A71**,3–8 (2015).
9. Sheldrick, G. M. Crystal structure refinement with SHELXL. *Acta Cryst.* **C71**, 3–8 (2015).
10. Müller, P. Practical suggestions for better crystal structures. *Cryst. Rev.* **15**, 57–83 (2009).
11. Spek, A. *Acta Crystallogr. Sect.* **C71**, 9–18 (2015).
12. Spek, A. L. Vol. *20*, PLATON, a multipurpose crystallographic tool, Utrecht University, Utrecht,The Netherlands, **2001**.
13. Spek, A. *Acta Crystallogr. Sect.* **D65**, 148–155 (2009).
